# Nanodrug delivery in reversing multidrug resistance in cancer cells

**DOI:** 10.3389/fphar.2014.00159

**Published:** 2014-07-10

**Authors:** Sonali Kapse-Mistry, Thirumala Govender, Rohit Srivastava, Mayur Yergeri

**Affiliations:** ^1^Department of Pharmaceutics, SVKM's Dr. Bhanuben Nanavati College of Pharmacy, University of MumbaiMumbai, India; ^2^Discipline of Pharmaceutical Sciences, School of Health Sciences, University of KwaZulu-NatalDurban, South Africa; ^3^Department of Biosciences and Bioengineering, Indian Institute of Technology BombayMumbai, India; ^4^Department of Pharmaceutical Chemistry, SVKM's Dr. Bhanuben Nanavati College of Pharmacy, University of MumbaiMumbai, India

**Keywords:** tumor microenvironment, drug efflux pumps, multidrug resistance, nanodrug delivery systems, theragnostic

## Abstract

Different mechanisms in cancer cells become resistant to one or more chemotherapeutics is known as multidrug resistance (MDR) which hinders chemotherapy efficacy. Potential factors for MDR includes enhanced drug detoxification, decreased drug uptake, increased intracellular nucleophiles levels, enhanced repair of drug induced DNA damage, overexpression of drug transporter such as P-glycoprotein(P-gp), multidrug resistance-associated proteins (MRP1, MRP2), and breast cancer resistance protein (BCRP). Currently nanoassemblies such as polymeric/solid lipid/inorganic/metal nanoparticles, quantum dots, dendrimers, liposomes, micelles has emerged as an innovative, effective, and promising platforms for treatment of drug resistant cancer cells. Nanocarriers have potential to improve drug therapeutic index, ability for multifunctionality, divert ABC-transporter mediated drug efflux mechanism and selective targeting to tumor cells, cancer stem cells, tumor initiating cells, or cancer microenvironment. Selective nanocarrier targeting to tumor overcomes dose-limiting side effects, lack of selectivity, tissue toxicity, limited drug access to tumor tissues, high drug doses, and emergence of multiple drug resistance with conventional or combination chemotherapy. Current review highlights various nanodrug delivery systems to overcome mechanism of MDR by neutralizing, evading, or exploiting the drug efflux pumps and those independent of drug efflux pump mechanism by silencing Bcl-2 and HIF1α gene expressions by siRNA and miRNA, modulating ceramide levels and targeting NF-κB. “Theragnostics” combining a cytotoxic agent, targeting moiety, chemosensitizing agent, and diagnostic imaging aid are highlighted as effective and innovative systems for tumor localization and overcoming MDR. Physical approaches such as combination of drug with thermal/ultrasound/photodynamic therapies to overcome MDR are focused. The review focuses on newer drug delivery systems developed to overcome MDR in cancer cell.

## Introduction

Cancer is a heterogeneous disease and use of multiple drugs simultaneously can result in drug resistance which is either intrinsic or acquired known as multidrug resistance (MDR). MDR renders cancer cells immune to standard treatments with many anticancer agents and is a major challenge in cancer therapy as it needs to address multiple phenotypes including MDR phenotypes. Tumor heterogeneity and tumor cell resistance to anticancer drugs thus remains key formidable challenges for effective targeting of drug delivery systems for successful chemotherapy. Drug resistance toward antineoplastic agents is a result of reduction in the effective concentration of drug in the cell prior to its interaction with the target or due to a combination of processes. The numerous mechanism of drug resistance reported includes (a) over expression of drug efflux pumps such as permeability glycoprotein (P-gp), multidrug resistance associated protein (MRP), and breast cancer resistance protein (BCRP) (b) alterations in lipid metabolism (ceramide pathway) (c) drug elimination by detoxification systems (d) drug test sequestration inside lysosomes and endosomes (e) reduced drug uptake due to altered surface receptors/carriers (f) inactivation of drugs via glutathione-mediated reduction (g) over expression of target enzymes such as up-regulated thymidylate synthase (h) altered drug targets such as topoisomerase II (i) increased DNA repair capacity (j) reduced ability to undergo apoptosis (k) hypoxia up-regulated expression of MDR-linked genes such as ABC transporters, Bcl-2 family genes, glutathione, metallothionein, etc. through activation of transcription factor HIF1 (l) chromosomal abnormalities in cancer cells lead to over-expression of anti-apoptotic genes (m) altered signal transduction pathways in cancer cells governed via integrin receptors, growth factor receptors etc. leads to blockage of apoptosis and expression of MDR-linked genes those involved in DNA repair and drug-efflux pumps (Broxterman et al., 2003).

**Figure 1:**
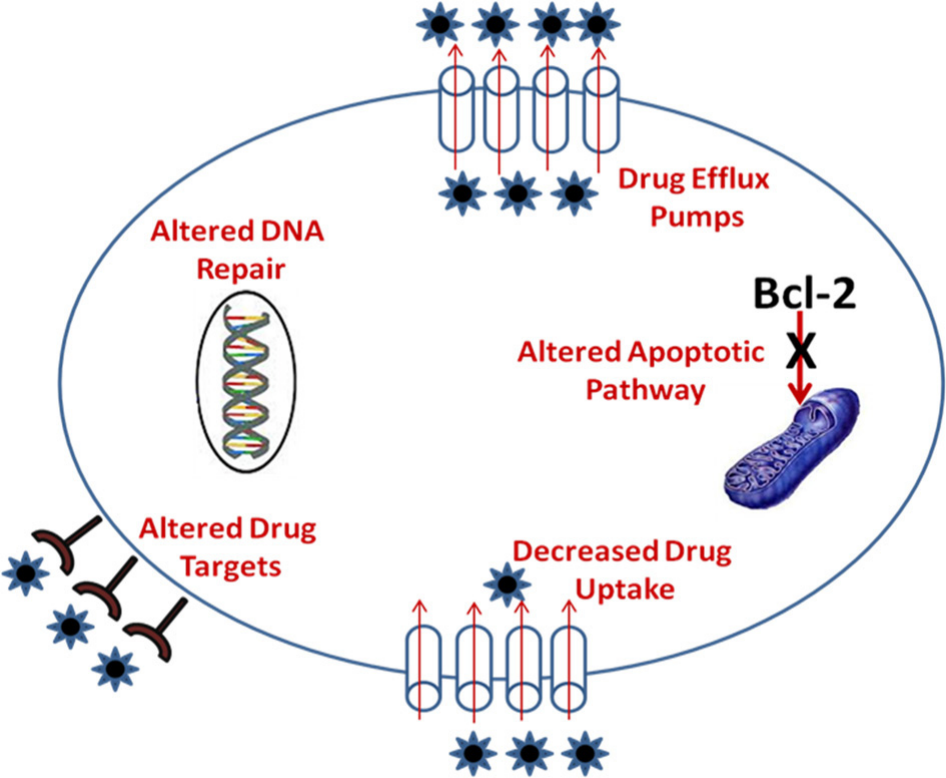
**Mechanism of multidrug resistance in tumor cell**.

Drug resistance mechanism of antineoplastic agents (Table 1) and mechanism of MDR in tumor cells is shown in Figure 1.

**Table 1:** **Drug resistance mechanisms of anticancer drugs**.

**Class**	**Example**	**Cytotoxicity mechanism**	**Molecules in resistance mechanism**
Intercalators	Doxorubicin Daunomycin	Topoisomerase II inhibitor, superoxides and free radicals	P-gp, Topoisomerase II, MRP, GST
Alkylators	Cyclophosphamide	DNA alkylation	O^6^-alkylguanine-DNA alkyltransferase, Glutathione, Aldehyde dehydrogenase
	Cisplatin	DNA alkylation	Glutathione, Metallothionein, DNA repair enzyme, multispecific organic anion transporter
Antimetabolites	BCNU	DNA alkylation	O^6^-alkylguanine-DNA alkyltransferase
	Methotrexate	Folic acid antagonist	Amplification of dihydrofolate reductase, MRP, decreased reduced folate carrier expression
Vinca alkaloids	5-Fluorouracil	Uracil analog	Amplification of thymidylate synthase
	Vinblastine	Tubulin	P-gp, MRP, Tubulin
	Vincristine	Polymerization inhibitor	Mutation
Epidophylotoxins	Etoposide	Topoisomerase II inhibitor	MRP, Glutathione, P-gp, Topoisomerase I
Taxanes	Paclitaxel	Microtubule assembly inhibitor	P-gp, altered α/β Tubulin

### Multidrug efflux pumps

Drug efflux pumps expressed on human cancer cells majorly contribute to MDR (Sharom, 1997). These efflux pumps belong to ATP-binding cassette (ABC) family and include (a) **P-gp** also known as multidrug resistance protein 1 (MDR1) or cluster of differentiation 243 (CD243) a ATP-binding cassette sub-family B member 1 encoded in human by *ABCB1* gene (b) **Multidrug Resistance Associated Protein 1 (MRP1)** a ATP-binding cassette sub-family C member 1 encoded in human by *ABCC1* gene, **Multidrug Resistance Associated Protein 2 (MRP2)** also called as canalicular multispecific organic anion transporter 1 (cMOAT) a ATP-binding cassette sub-family C member 2 encoded in human by *ABCC2* gene (c) **BCRP** also known as cluster of differentiation (CDw338) a member of white sub-family and ATP-binding cassette G member 2 encoded in human by *ABCG2* gene (Ozben, 2006).

### P-glycoprotein (P-gp)

P-gp is the first member of ABC super family and is an ATP-powered drug efflux pump membrane transporter (Fardel et al., 1996; Sharom, 1997). Over-expression of P-gp in mammalian and human cancer cells results in MDR. P-gp has two isoforms expressed in human, class I and III isoforms are drug transporters (*MDR1/ABCB1*) while class II isoforms export phosphatidylcholine into bile (*MDR2/3/ABCB4*) (Sharom, 1997). P-gp encoded by MDR1 gene is present in human tissues including liver, kidney, pancreas, small and large intestine while P-gp encoded by MDR2 gene is present at high levels only in liver (Fardel et al., 1996). Carcinoma of colon, kidney, adrenal gland, pancreas, and liver express high P-gp levels while intermediate P-gp levels are expressed in neuroblastomas, soft tissue carcinomas, hematological malignancies including CD34-positive acute myeloid leukemias, etc. with low P-gp levels expressed in malignancies of lung, esophagus, stomach, ovary, breast, melanomas, lymphomas, multiple myelomas, and acute promyelocytic leukemia but may display elevated P-gp levels after chemotherapy due to acquired drug resistance (Velingkar and Dandekar, 2010). P-gp interacts with structurally diverse substrates such as anticancer drugs, HIV protease inhibitors, analgesics, calcium channel blockers, immunosuppressive agents, cardiac glycosides, antihelminthics, antibiotics, H_2_-receptor antagonists, steroids, fluorescent dyes, linear and cyclic peptides, ionophores, peptides, lipids, small cytokines such as interleukin-2, intereukin-4, and interferon-γ, MDR chemosensitizers, and many more (Velingkar and Dandekar, 2010).

### First generation inhibitors

These are non-selective, less potent with poor, and low binding affinity; requiring high doses to achieve plasma levels to reverse MDR, resulting in unacceptable patient toxicity. They are substrates for P-gp and act as competitive inhibitors thereby requiring high serum concentrations of chemosensitizers to produce adequate intracellular concentrations of cytotoxic drug due to which these inhibitors are unsuccessful in clinical trials (Dantzig et al., 2003). First generation inhibitors include Verapamil, Trifluoperazine, Cyclosporine-A, Quinidine and Reserpine, Vincristine, Yohimbine, Tamoxifen, and Toremifene. Due to unpredictable pharmacokinetic interactions of these substrates in presence of chemotherapy agents several novel chemosensitizers analogs were developed with less toxicity and greater potency.

### Second generation inhibitors

Structural modifications of first generation inhibitors resulted in more potent second generation P-gp modulators with better pharmacological profile, reduced toxicity, and better tolerability. They significantly inhibit metabolism and excretion of cytotoxic agents leading to unacceptable toxicity necessitating chemotherapy dose reductions. Successful treatment of refractory cancers and reversal of MDR in clinical trials have been possible by co-administration of these modulators with chemotherapy agents. Modulators include Dexverapamil, Dexniguldipine, Valspodar (PSC 833), and Biricodar citrate (VX-710).

### Third generation inhibitors

They have high potency and specificity for P-gp transporters over second generation agents. They do not interfere with cytochrome P450 3A4 unaffecting drug pharmacokinetics with no dose alterations in chemotherapy. They include Tariquidar-XR9576, Zosuquidar-LY335979, Laniquidar-R101933, ONT-093 (substituted diarylimidazole), Elacridar-GF120918, OC 144-093, Mitotane (NSC-38721), Annamycin, and R101933 (Ozben, 2006). Most promising Tariquidar (non-transported P-gp inhibitor) which inhibits ATPase by interaction with protein is currently in phase III trials for non-small cell lung cancer but still suspended due to unfavorable toxicity. Clinical trial studies revealed that Tariquidar, LY335979, R101933, and ONT-093 can be administered with therapeutic doses and minimal interference with pharmacokinetics of cytotoxic agents. They have shown promise in clinical trials and continued development of these agents may establish the true therapeutic potential of P-gp mediated MDR reversal.

Tariquidar a third generation inhibitor with no limitations of first and second generation inhibitors, have highest specificity which specifically and potently inhibits P-gp. Inhibition of ATPase activity of P-gp suggests that the modulating effect is derived from inhibition of substrate binding, inhibition of ATP hydrolysis and or both (Fox and Bates, 2007). Clinical trials of third-generation inhibitors (Thomas and Coley, 2003) showed better tolerability of Tariquidar with no significant pharmacokinetic interaction with chemotherapy. This makes Tariquidar an ideal agent for demonstrating P-gp inhibition activity in cancer. Targeted delivery of paclitaxel and tariquidar co-encapsulated in biotin functionalized PLGA nanoparticles revealed significantly higher cytotoxicity *in vitro* and greater tumor growth inhibition *in vivo* in drug-resistant tumor mouse model compared to paclitaxel nanoparticles alone with promising results in clinical trials (Patil et al., 2009b).

### Tumor microenvironment and MDR

Tumors are core-shell structures with hypoxic core surrounded by tissues and proliferative cells. Tumor microenvironment is made of complex tissues containing extracellular matrix, activated fibroblasts, immune cells, pericytes, adipocytes, epithelial cells, glial cells, vascular and lymphatic endothelial cells, and numerous proteins (van Kempen et al., 2003; Weber and Kuo, 2012). The proliferative cells are highly vascularized, unorganized and discontinuous resulting in enhanced permeability and retention (EPR) effect widely exploited for passive targeting. The major factors contributing to tumor progression and metastasis, enhanced drug resistance, poor prognosis, and response to therapies includes cell mobility, survival potential, capacity to degrade extracellular tissue matrix, and ability to adjust in new tissue environment (Otranto et al., 2012; Singh and Kaur, in press). All solid tumor microenvironment possess the following characteristics (Milane et al., 2011) (Table 2) (a) leaky and unorganized tumor vasculature (b) hypoxia region (c) up-regulation of oncogenes (d) DNA repair mechanisms (e) down regulation of tumor suppressors and cell cycle regulation (f) increased growth factor receptors (g) low nutrients. Tumor microenvironment significantly contributes to drug resistance by reducing drug accessibility to tumor cells and reduces the oxygen radicals generated by antitumor drugs (Otranto et al., 2012; Singh and Kaur, in press). Hypoxia and acidity with low nutrient levels remains the two key factors characterizing tumor microenvironment (Schornack and Gillies, 2003; Wouters et al., 2003). Tumor hypoxia is low oxygen regions with partial oxygen pressure (pO_2_) levels below 10 mm-Hg where normal tissues range from 24 to 66 mm-Hg (Rofstad, 2000). Hypoxia microenvironment is characterized by low pH (acidic cell environment) and can be associated with activation of proteases that contributes to metastasis, low glucose levels, high interstitial fluid pressure due to leaky vasculature, impaired lymphatic drainage, and high levels of P-gp (Tomida and Tsuruo, 2002). Hypoxia Inducible Factor (HIF) (Harris, 2002) is another mechanism that induces MDR and metastasis by up-regulating target genes by binding to hypoxia-response element (HRE) in the target. HIF-1 is a transcription factor activated in hypoxia. While tumor acidic pH results in poor tumor perfusion due to abnormal vascularization, hypoxia, and metabolic abnormalities are associated with cell growth and increased capacity for transmembrane pH regulation (Simon et al., 1994). Both pO_2_ and pH are important determinants of tumor growth, metabolism, and response to variety of therapies (Fukumura and Jain, 2007). Acidic extracellular pH restricts uptake of weak base drugs such as Adriamycin, Doxorubicin, and Mitoxantrone. Both hypoxia and acidic pH contributes to growth and tumor metastasis (Harris, 2002). Hypoxia upregulates various angiogenic growth factors including Vascular Endothelial Growth Factor (VEGF), Angiopoietin (Ang) 2, Platelet Derived Growth Factor (PDGF), Placenta Growth Factor (PGF), Transforming Growth Factor α (TGFα), Interleukin (IL)-8, and Hepatocyte Growth Factor (HGF) of which Hypoxia Inducible Factor 1α (HIF1α) is considered the master regulator of oxygen homeostasis.

**Table 2:** **Tumor microenvironment characteristics contributing toward MDR**.

**Increased levels**	**Decreased levels**
Oncogenes	Tumor suppressors
Growth factors/receptors	Oxidative phosphorylation
Nutrient importers	pH
ABC transporters	Cell cycle regulation
Aerobic glycolysis	Increased apoptosis
Interstitial fluid pressure	
DNA repair	
Detoxification enzymes	

## Strategies to overcome MDR in cancer cells

### Modification of chemotherapy regimens

Chemotherapy regimen includes “induction regimen” and “maintenance regimen” refers to initial disease treatment and ongoing chemotherapy to reduce chances of cancer recurrence or prevent growth of an existing cancer, respectively. Combination chemotherapy utilizes synergistic effect of multiple antineoplastic drugs acting through different mechanisms, but due to their different dose-limiting adverse effects they are given together in chemotherapy regimens. Chemotherapy regimen needs to balance efficacy and toxicity through proper dosing schedule. Dose-dense regimens have more toxic effects than standard regimen causing treatment delays and toxicity with few survival improvements and early treatment discontinuation. A dose-dense approach is more effective than standard approach, as it hampers formation of blood vessels that feed tumors and tumor shrinkage following treatment promoting tumor dormancy by maintaining tumor size and preventing outgrowth. Chemotherapy regimens are identified by acronyms, identifying the drug combination agents. E.g., (i) Breast cancer: AC (Adriamycin, Cyclophosphamide), CAF (Cyclophosphamide, Adriamycin, Flurouracil), EC (Epirubicin, Cyclophosphamide), FEC (Flurouracil, Epirubicin, Cyclophosphamide); (ii) Colorectal cancer: FL (Fluorouracil, Leucovorin), FOLFOX (Fluorouracil, Leucovorin, Oxaliplatin), FOLFIRI (Fluorouracil, Leucovorin, Irinotecan). Chemotherapy regimen is based on the assumption that the mutations conferring drug resistance will not convey resistance to all the agents in the regimen and high-dose chemotherapy regimens could be given to cancer patients. Such approach assumes that despite resistance to standard doses of anticancer drugs, a dose-response relationship exists for tumors and high doses of chemotherapy might overcome the resistance.

### Inactivation of MDR-associated genes by targeting specific mRNA for degradation

Strategies to overcome multi drug resistance by silencing the expression of gene encoding P-gp efflux transporter, i.e., MDR-1 or Survivin through RNA interference (RNAi) or small interfering RNA (siRNA) has been explored. Transient RNAi mediated silencing can be achieved by siRNA or stable RNAi-mediated gene silencing through short hairpin RNA (shRNA) transfection. The siRNAs assembles into endoribonuclease inside the cells containing complexes known as RNA-Induced Silencing Complexes (RISCs) which guides the RISCs to complementary RNA molecules, cleaving and destroying the target RNA. Antisense oligonucleotides and catalytic RNAs have been successful in inhibiting P-gp, MRP, and BCRP expression and sensitized drug-resistant cells (Nadali et al., 2007; Ren et al., 2008). *In vitro* and *in vivo* studies with biotin-functionalized nanoparticles co-encapsulating paclitaxel and P-gp targeted siRNA partially overcame tumor drug resistance (Patil et al., 2010). Two groups, Nieth et al. (2003) and Wu et al. demonstrated that RNAi knock downs the MDR1/P-gp encoding mRNA and reverse the MDR phenotype of cancer cells. They further chemically synthesized siRNA to transiently down regulate MDR1/P-gp mRNA and protein expression. To overcome MDR in cancer, Lage (2009) developed anti-ABC transporter shRNA expression vectors with high potential to overcome MDR through silencing specific ABC transporter transcripts. These studies revealed total knock down of mRNA and protein by inhibition of P-gp and reversal of drug-resistant phenotype. Efficiency of RNAi to overcome MDR *in vivo* were performed by transfecting MDR cancer cells with anti-MDR shRNA expression plasmids. Treatment of these cells grown as xenografts in nude mice with vincristine revealed tumor growth inhibitin by 42% for the shRNA expressing tumors. Tumor growth inhibition by 80-fold was observed in cells transfected with anti-MDR1/P-gp shRNA expressing retroviruses implanted in nude mice (Milane et al., 2011).

### Monoclonal antibodies for P-gp

Monoclonal antibodies (MAbs) have potential for targeting P-gp and kill MDR tumor cells. Anti-P-gp MAbs such as MRK-16 and MRK-17 along with chemosensitizers reverses P-gp mediated MDR and conjugated MAbs such as bispecific antibody, immunotoxin and radioisotope conjugates enhance anti-tumor activity. Combination of MRK-16 with Cyclosporin-A or PSC-833 reversed Doxorubicin resistance in K562/ADM cells and inhibited tumor growth in athymic mice bearing HCT-15/ADM2-2 xenografts. MRK-16 increased Cyclosporine-A accumulation in MDR cells but not affected intracellular PSC-833 accumulation in MDR cells, instead Cyclosporin-A and PSC-833 increased MRK-16 binding to P-gp revealing a synergistic MDR reversal activity. MAbs with other anti-P-gp MAbs such as UIC2, 4E3, and series of HYB antibodies have potential to inhibit drug transport (Tomida and Tsuruo, 2002).

### Development of new anticancer drugs that are not substrates of P-gp

Drug analogs such as Taxane analogs DJ-927 (Phase I), BMS-184476 (Phase I), RPR 109881A (Phase II), Ortataxel (Phase II), Trabectedin-ET-743 (Phase II and III) are not recognized by P-gp transporter and are evaluated in clinical trials for their broad spectrum activity in sensitive and resistant tumor cell lines to overcome MDR (Dong and Mumper, 2010). DJ-927 was more potent and cytotoxic than paclitaxel and docetaxel when compared *in vitro* and *in vivo* in various P-gp expressing tumor cell lines with high intracellular accumulation in P-gp positive cells. The expression of P-gp levels or P-gp modulators did not affect the tumoricidal efficacy of DJ-927. Phase I study of DJ-927 in combination with capecitabine was acceptable with no pharmacokinetic drug interactions in patients with advanced solid tumor malignancies and is recommended for further clinical studies. Preclinical studies showed that BMS-184476 was more potent than paclitaxel against taxane sensitive and resistant tumors. The P-gp over-expressing human colon cancer cell line (HCT-116/MDR) was 62-fold more resistant to paclitaxel and 15-fold resistant to BMS-184476. Also the human ovarian cancer cells with acquired taxane resistance expressed 9-fold resistance to BMS-184467 and 32-fold to paclitaxel. Studies of BMS-184476 against human tumor xenografts with both acquired and primary taxane resistance models revealed superiority of BMS-184476 (Yared and Tkaczuk, 2012).

### Inhibitors of ABC transporters to reverse MDR

Inhibition of ABC transporters should reverse MDR by increasing intracellular drug concentrations in tumor cells and restore drug sensitivity. These inhibitors transport themselves and then act as competitive antagonists while others are not transported but affect transporter function (Dong and Mumper, 2010). Preclinical trials of first and second generation ABC transport inhibitors were not successful. They failed in clinical trials due to their non-specificity, high concentrations to inhibit activity, undesirable drug interactions due to co-administration of inhibitors and anticancer drugs (e.g., verapamil and doxorubicin), substrates of cytochrome P-450 and increased toxicity of anticancer drugs. Clinical trials of third generation inhibitors with LY335979 (Zosuquidar), GF120918 (Elacridar), R101933 and XR9576 (Tariquidar) are ongoing. Tariquidar in phase I studies revealed high potency in *in vitro* and *in vivo* studies. LY335979 prolonged survival by reducing tumor growth in mice with drug resistant tumors, GF120918 enhanced topotecan bioavailability in mice by sensitizing human MDR sarcoma MES-Dx5 cells. Although phase I and II clinical trials of third generation inhibitors are promising but are limited to unpredictable pharmacokinetic drug interactions, simultaneous involvement of several drug transporters and variability in drug transporter expression levels among individuals restricts restoration of drug sensitivity of such modulators in clinic (Wu et al., 2008).

### Nanotechnology based approaches to overcome MDR

Nanocarriers to overcome MDR are extensively discussed in section “Nanocarriers as potential drug delivery systems in cancer therapy.” Nanocarriers have been developed encapsulating anticancer drugs as P-gp substrates and/or with P-gp substrates.

### Inhibition of MDR using peptides

Synthetic P-gp peptides derived from fragments of extracellular loops of murine P-gp coupled with polyethylene glycol and loaded in Doxorubicin liposomes have shown MDR reversal with 83% increase in survival time of mice inoculated with P388R cells. Antitumor effect of peptide-conjugated Doxorubicin in human erythroleukemic (K562/ADR) resistant cells showed dose-dependent inhibition of cell growth against K562/ADR cells as compared with Doxorubicin alone (Dong and Mumper, 2010).

## Nanocarriers as potential drug delivery systems in cancer therapy

Nanovehicles such as polymeric nanoparticles, solid lipid nanoparticles, magnetic nanoparticles, dendrimers, liposomes, micelles, quantum dots, etc. are extensively explored for cancer diagnosis, treatment, imaging, and as ideal vectors to overcome drug resistance by diverting ABC-transporter mediated drug efflux mechanisms. The major classes of nanocarriers utilized for chemotherapeutic drug delivery are listed in Table 3 (Ayers and Nasti, 2012).

**Table 3:** **Chemotherapeutic nanodrug delivery systems**.

**Nanocarriers**	**Properties**	**Characteristics**
Solid lipid nanoparticle (SLNs)	Release drug in acidic microenvironment of multidrug resistance cells	Delivers anticancer drugs to overcome P-gp mediated multidrug resistance
Polymeric nanoparticles (NPs)	Versatile platform for controlled, sustained, and targeted delivery of anticancer agents including small molecular weight drugs and macromolecules (genes and proteins)	Enhanced drug accumulation, reduction in tumor size/volume, increased animal survival rate in rat models, minimal cytotoxicity in cancer cell lines, high transfection activity, potential to overcome multidrug resistance
Liposomes (LIPO)	Made of lipid bilayers encapsulating both hydrophobic and hydrophilic drugs, stealth liposomes are surface coated with PEG	Long-circulating, prevents non-specific interactions, preferential accumulation in tumor tissues via enhanced permeability, and retention effect to overcome drug resistance
Micelles (MI)	Small size, high payload capacity, greater solubilization potential for hydrophobic drugs, improved stability, long circulation	Selective targeting, P-gp inhibitory action, altered drug internalization, and sub-cellular localization properties
Mesoporous silica nanoparticles (MSNPs)	Inorganic nanocarriers with tunable size and shape, high drug loading due to high pore volume and surface area, multifunctionalization for targeted, and controlled delivery	Enhanced cellular uptake and bioavailability, circumvents unwanted biological interactions, delivers therapeutics at cellular levels for therapeutic, and imaging in cancer
Inorganic nanoparticles (a) Iron oxide magnetic nanoparticles	Unique optical, electrical, magnetic and/or electrochemical properties, inert, stable, ease of functionalization	Circumvents drug resistance associated with over expression of ATP-binding cassette transporters, increased intracellular drug retention, enhanced loss of cell viability
(b) Gold nanoparticles (AuNPs)	Shape and size dependent on electronic characteristics, versatile drug delivery system due to tunable optical properties	Induces cellular DNA damage
(c) Quantum dots (QD)	Semiconductor inorganic fluorescent nanocrystals, small (1–20 nm), and uniform size, high surface to volume ratio, surface conjugation with multiple ligands, biocompatible, fluorescence properties help real time tracks within target cells	Release of toxic compounds (cadmium) and generation of reactive oxygen species can result in long term toxicity

### Polymeric nanoparticles

Polymeric nanoparticles have emerged as a versatile nanotechnology platform for controlled, sustained and targeted delivery of anticancer agents including small molecular weight drugs and macromolecules such as genes and proteins (Wang et al., 2009; Sahay et al., 2010; Tang et al., 2010). A significant reduction in tumor size and increased animal survival rate in rat xenograft glioma model with indomethacin loaded nanocapsules was observed by Bernardi et al. (2009). PLGA loaded cystatin nanoparticles and PLGA loaded cytokeratin specific monoclonal antibody nanoparticles neutralized excessive proteolysis preventing metastatic and invasive potential of breast tumor cells (Kos et al., 2009). Paclitaxel loaded PLA immuno-nanoparticles covalently coupled with humanized monoclonal antibodies (antiHER2) actively targeted tumor cells over expressing HER2 receptors (Cirstoiu-Hapca et al., 2009). Folic acid receptors over-expressed on human cancer cells (Antony, 1996; Wang et al., 2010) are studied in tumor models including mouse M109 carcinoma, KB human epidermal carcinoma cell line and mouse J6456 lymphoma (Alberto et al., 2004). Paclitaxel loaded PLA-PEG-ligand conjugated nanoparticles functionalized with biotin and folic acid enhanced drug accumulation in MCF-7 tumor xenograft model (Patil et al., 2009b). Lee et al. found that folic acid conjugated chitosan nanoparticles showed higher transfection activity than unmodified chitosan nanoparticles (Lee et al., 2006). Wang et al. (2010) observed 35% reduction in tumor growth, inhibition of P-gp and mdr1 gene levels in KB-A-1 cells implanted in Balb/c-nu/nu mice targeted by folic acid conjugated antisense oligodeoxynucleotides-hydroxypropyl-chitosan nanoparticles compared to bare antisense oligodeoxynucleotides to overcome tumor drug resistance. Folate functionalized PLGA nanoparticles loaded with anti-cancer drug nutlin-3a and chemosensitizer Curcumin enhanced therapeutic potential of nutlin-3a by modulating MDR of Y79 retinoblastoma cell through Curcumin and enhanced the anticancer activity of nutlin-3a in drug resistance Y79 cells. Dual drug loaded nanoparticles revealed better therapeutic efficacy with enhanced expression or down regulation of proapoptotic/antiapoptotic proteins and down-regulation of Bcl2 and NF-κB protein. Study demonstrated the role of Curcumin as MDR modulator to enhance the therapeutic potential of nutlin-3a for targeting MDR cancer (Das and Sahoo, 2012).

Silencing P-gp expression by RNAi with reduction-sensitive linear cationic click polymer nanoparticles (RCPNs) loaded with plasmid iMDR1-pDNA for gene delivery revealed higher transfection efficiency and lower cytotoxicity than PEI/DNA nanoparticles against human breast cancer MCF-7 cells and drug-resistant MCF-7/ADR cells (Gao et al., 2011). Vincristine sulfate loaded nanoassemblies enhanced cytotoxicity by 36.5-fold and cellular accumulation by 12.6-fold in MCF-7 and P-gp over expressing MCF-7/ADR cells compared to vincristine sulfate solution and overcome MDR by clathrin and caveolae mediated endocytosis pathways (Zhang et al., 2011b). Co-delivery of Paclitaxel and survivin shRNA nanoparticles lowered IC_50_ by 360-fold in Paclitaxel resistant lung cancer cells against A549/T cells compared to free Paclitaxel and enhanced efficacy with Paclitaxel induced apoptosis and cell arrest in G2/M phase. Nanoparticles facilitated drug accumulation in tumor cells and down-regulated of survivin shRNA into nuclei of lung cancer cells lowering the apoptosis threshold of drug resistant cells and renders chemotherapeutic agents more effective to overcome MDR (Shen et al., 2012). Docetaxel loaded poly(ε-caprolactone)/Pluronic F68 nanoparticles increased drug uptake and enhanced cytotoxicity in docetaxel-resistance human breast cancer cell line and MCF-7 TAX30 compared to poly caprolactone nanoparticles indicating its potential to overcome MDR (Mei et al., 2009). Lipid/particle assemblies (LNPs) loaded with Doxorubicin in DMAB-modified PLGA nanoparticles coated with DPPC lipid shell significantly increased accumulation and improved nucleus targeting in MCF-7 cells and P-gp over expressing resistant MCF-7/ADR cells relative to free drug and reversed the transporter-mediated drug resistance in human breast cancer. Cytotoxicity (IC_50_) of Doxorubicin loaded-LNPs was 30-fold lower than free Doxorubicin in MCF-7/ADR, indicating intracellular retention of Doxorubicin and bypassing drug resistance (Li et al., 2012a). Co-delivery of MDR1 siRNA via lipid-modified dextran-based polymeric nanoparticles with Doxorubicin increased intracellular drug concentration in MDR cell nucleus and efficiently suppressed P-gp expression in drug resistant osteosarcoma cell lines (KHOS_R2_ and U-2OS_R2_) (Susa et al., 2010). Pramanik et al. developed composite nanoparticles of Doxorubicin with Curcumin a potent MDR inhibitor to overcome Doxorubicin resistance in multiple *in-vivo* models such as multiple myeloma, acute leukemia, prostate and ovarian cancers. Composite nanoparticles revealed no cardiac toxicity or bone marrow suppression compared to free Doxorubicin (Pramanik et al., 2012). P-glycoprotein mediated efflux can be effectively circumvented by co-administration of P-gp inhibitor/s and anticancer drug/s in nanoparticles which evades P-gp recognition at cell membrane and delivers drug in the cell cytoplasm or nucleus thereby sustaining delivery of the drug inside the cell. Chavanpatil et al. encapsulated paclitaxel a P-gp substrate and verapamil a P-gp inhibitor in PLGA nanoparticles to circumvent P-gp-mediated drug efflux in MDR tumor cells (Chavanpatil et al., 2006). Doxorubicin loaded aerosol OT (AOT)-alginate nanoparticles enhanced the cellular delivery and therapeutic efficacy of P-gp substrates in P-gp over expressing cells (Chavanpatil et al., 2007). Novel polymer-lipid hybrid nanoparticle loaded with doxorubicin and chemosensitizer (GG918) evaluated in human MDR breast cancer cell line (MDA435/LCC6/MDR1) demonstrated nuclear drug localization and anticancer activity toward MDR cells, while co-administration of the single-agents loaded nanoparticles resulted in high cellular internalization but were ineffective (Wong et al., 2006). Encapsulation of paclitaxel with P-gp modulator tariquidar in poly (D, L-lactide-co-glycolide) nanoparticles functionalized with biotin revealed higher *in-vitro* cytotoxicity and increased intracellular accumulation compared to paclitaxel nanoparticles alone in drug-resistant tumor cells to overcome tumor drug resistance through biotin receptor-mediated endocytosis (Patil et al., 2009a).

### Solid lipid nanoparticles (SLNs)

*In-vitro* cytotoxicity in resistant P388/ADR cell line and *in-vivo* studies in P388/ADR leukemia mouse model revealed lowering of IC_50_ value by 9-fold and greater median survival time about 20 days (3.5 mg/kg dose) with Doxorubicin SLN compared to Doxorubicin solution. While comparable cell uptake and IC_50_ values were obtained with both Idarubicin SLN and free Idarubicin in P-gp over expressing P388/ADR and HCT-15 cells mouse tumor models. Study revealed the potential of Doxorubicin SLN in overcoming P-gp-mediated MDR both *in-vitro* in P388/ADR leukemia cells and *in-vivo* in murine leukemia mouse model (Ma et al., 2009). Greater accumulation of Doxorubicin SLN in MCF-7/ADR cells over expressing P-gp with enhanced apoptotic cell death and decreased cell viability compared to plain Doxorubicin revealed the potential of Doxorubicin SLNs to overcome chemoresistance in adriamycin-resistant breast cancer cell line. Decrease in the intensity of 116-kDa PARP band (DNA repair enzyme activated by DNA damage and used as apoptosis biochemical marker) in MCF-7/ADR cells treated with 3 μM either of Doxorubicin or SLN alone or Doxorubicin SLN indicated efficiency of Doxorubicin SLN to cause cell death through induction of apoptosis in Doxorubicin resistant cancer cells. Cellular uptake of Doxorubicin SLN was 17.1-fold and 21.6-fold higher than Doxorubicin alone implying potential of SLNs in diminishing P-gp mediated drug efflux (Kang et al., 2010). SLNs being easily internalized enhanced cellular uptake and cytotoxicity of Doxorubicin and Paclitaxel loaded solid lipid nanospheres in human promyelocytic leukemia cells (HL60) and human breast carcinoma cells (MCF-7) compared to free drug solutions. Paclitaxel solid lipid nanospheres were 100-fold more effective than free Paclitaxel in MCF-7 cells with low sensitivity on HL60 cells. Doxorubicin SLN enhanced cytotoxicity and sensitivity on MCF-7 cells (10-fold) and on HL60 cells (>40-fold) with IC_50_ at 1 ng/ml compared to Doxorubicin solution reducing drug cell resistance. Such increased cytotoxicity of Doxorubicin nanocarriers compared to solution has been earlier reported with polymeric nanoparticles, micelles and liposomes. Enhanced intracellular accumulation and cytotoxicity of Doxorubicin loaded pluronic copolymer micelles have been reported by Kabanov and coworkers. Couvreur reported that Doxorubicin loaded polyalkylcyanoacrylate nanoparticle were more cytotoxic than Doxorubicin solution against P388 leukemia cells overcomed MDR and decreased cell viability against resistant MCF-7 cell-lines (Couvreur and Vauthier, 1991). Paclitaxel SLN enhanced cytotoxicity (100-fold) at concentration >5 ng/ml on HL60 cells and at 1 ng/ml on MCF-7 cells (Miglietta et al., 2000). Polymer-lipid hybrid nanoparticle with Doxorubicin and chemosensitizer (GG918) or their combination revealed high Doxorubicin uptake in human MDR breast cancer cell line (MDA435/LCC6/MDR1) compared to co-administration of two single-agent/s loaded hybrid nanoparticles (Wong et al., 2006). Tween^®^80 coated Edelfosine lipid nanoparticles revealed antiproliferative effect due to P-gp inhibitory action on C6 glioma cell lines and significantly reduced the tumor growth within 14 days post treatment in nude mice bearing C6 glioma xenograft tumor (Mendoza et al., 2011). Paclitaxel and Doxorubicin SLN exhibited higher cytotoxicity in human breast tumor drug sensitive MCF-7 and drug resistant MCF-7/ADR cells compared to Taxol and Doxorubicin solution. Paclitaxel and Doxorubicin loaded SLN revealed 31.0- and 4.3-fold reversal in drug resistance of MCF-7 cells compared to MCF-7/ADR cells respectively (Miao et al., 2013). Doxorubicin-mitomycin co-loaded stealth polymer-lipid hybrid nanoparticles enhanced efficacy in sensitive and MDR human mammary tumor xenografts with 3-fold increase in life span, 10–20% tumor cure rate, inhibition of tumor angiogenesis with no severe tissue toxicity compared to liposomal Doxorubicin (Prasad et al., 2013). P-glycoprotein efflux at the brain limits entry of Docetaxel for cancer treatment. Folic acid modified solid lipid nanoparticles loaded with docetaxel and ketoconazole (P-gp inhibitor) evaluated in brain endothelial cell lines for cytotoxicity and cell uptake revealed a brain permeation coefficient 44 times higher than that of Taxotere^®^ (Venishetty et al., 2013). Docetaxel loaded hepatoma-targeted SLNs revealed high cellular uptake by hepatoma cells, better biodistribution and enhanced antitumor efficacy due to increased drug accumulation and cytotoxicity in murine model bearing hepatoma and hepatocellular carcinoma cell line BEL7402 compared to Taxotere^®^ or non-targeted SLNs for treatment of advanced and metastatic hepatocellular carcinoma (Xu et al., 2009). A lipophilic paclitaxel derivative (2′-behenoyl-paclitaxel) (C22-PX) conjugated in lipid nanoparticle for metastatic breast cancer improved antitumor efficacy, tumor retention, better tolerability, and higher plasma levels compared to Taxol in a subcutaneous 4T1 mouse mammary carcinoma model (Ma et al., 2013).

### Liposomes

Liposomal anthracyclines approved by US FDA for treatment of AIDS-related Kaposi's sarcoma are pegylated liposomal doxorubicin (Doxil^®^/Caelyx^®^) and liposomal daunorubicin (DaunoXome^®^) which preferentially accumulates in tumor tissues via EPR effect to overcome drug resistance or accumulates within extracellular space of tumor stroma and leaks into tumor environment which provides pharmacologic advantage for liposomes over free drug to overcome drug resistance (Table 4). Currently liposomes of Paclitaxel, Camptothecins and Vincristine are in clinical development. Liposomal strategies to enhance drug bioavailability and efficacy in drug-resistant cancer include (i) liposomes modified for controlled release (ii) ligand targeted liposomes such as immunoliposomes for intracellular drug delivery in tumor cells.

**Table 4:** **Marketed liposomal delivery systems to overcome drug resistance**.

**Marketed liposomes**	**Rationale**	**Mechanism**
Pegylated liposomal Doxorubicin (Doxil^®^/Caelyx^®^)	Long-circulating liposomes preferentially accumulates in tumor tissue	Increased tumor exposure
Non-pegylated liposomal Doxorubicin (Myocet^™^)	Liposome leads to altered biodistribution, reduced drug toxicity profiles with new chemotherapeutics combinations to overcome drug resistance	Reduced toxicity profile
Liposomal Daunorubicin (DaunoXome^®^)	Liposomal encapsulated Doxorubicin is less cardiotoxic than unencapsulated Doxorubicin and can be safely used in concurrent combination with other cardiotoxic chemotherapy drugs such as Trastuzumab Minimal side effects allow substitution with Doxorubicin in same treatment regimen improving safety with no loss of efficacy	

Liposomes directly interact with P-gp and inhibit P-gp through endocytosis. Liposome co-encapsulating Doxorubicin and Verapamil conjugated with human transferrin (Tf) showed greater cytotoxicity, selective targeting and reversal of P-gp mediated drug resistance in resistant leukemia K562 cells than non-targeted co-loaded liposomes. Doxorubicin liposomes increased cytotoxicity on HL60 cells and Vincristine resistant HL60 cells due to rapid internalization and drug release inside the cells (Gokhale et al., 1996). Robert Lee et al. found that uptake of folate-PEG-liposomal Doxorubicin by KB cells was 45-fold higher than non-targeted liposomal Doxorubicin (Lee and Low, 1995). Liposomes overcome drug resistance due to endothelial P-gp efflux mechanism at blood-brain and blood-tumor barriers in brain tumors where the barriers allows extravasation of long circulating liposomes and circumvent drug resistance with stabilized liposomal Doxorubicin in rat intracranial sarcoma model (Siegal et al., 1995) and rat intracranial 9L gliosarcoma model (Zhou et al., 2002).

### Modified liposomes to overcome drug resistance

New liposomal systems developed for treatment of drug-resistant cancers are listed in Tables 5, 6.

**Table 5:** **Modified liposome approaches to overcome multidrug resistance**.

**Modified liposomes**	**Mechanism of action**
Anionic liposomes	Anionic lipids (Cardiolipin and Phosphatidylserine) inhibits P-gp by direct interaction with membrane lipids, enhance cellular absorption, and cellular toxicity compared to free drugs
Inhibitory phospholipids	Inhibits P-gp to overcome multidrug resistance
Stimuli responsive liposomes	Modified liposomes which release drug in target tissue upon hyperthermia treatment/temperature change, pH change, or other stimuli
Liposomes in combination with resistance inhibitors	Liposome inhibits P-gp and successfully delivers chemotherapeutic to cancer cells and increase drug therapeutic index
Liposomes encapsulating drug analogs	Liposomes delivers hydrophobic drugs that are not substrates for P-gp or not effluxed by P-gp
Gene therapy approaches	Non-viral delivery of nucleic acid to tumor cells circumvents drug resistance, non-viral delivery of resistance genes to normal tissues gives chemoprotection

**Table 6:** **Immunoliposomes and ligands-liposomes to overcome drug resistance**.

**Liposomes**	**Mechanism**
Immunoliposomes for growth factor receptors	Targeting growth factor receptors with liposomes encapsulating monoclonal antibodies (MAbs) for targeting undergo endocytosis pathways to overcome drug efflux pumps
Immunoliposomes for endothelial receptors	Unlike cancer cells, endothelial cells do not develop multidrug resistance
Immunoliposomes for P-gp	Multidrug resistance is reversed with MAbs against P-gp

### Micelles

Micelles are efficient drug carriers with potential P-gp inhibitory action, altered drug internalization, subcellular localization and selective targeting. Seven anti-tumor drugs loaded polymeric micelles in clinical trials are Genexol^®^-PM, NK105, NC-6004, NC-4016, NK012, NK911 and SP1049C (Gong et al., 2012). Micelles overcome drug resistance by combination of mechanisms including EPR effect, active internalization, endosomal-triggered release and drug escape. Folate decorated pH-sensitive Doxorubicin micelles showed high drug concentration in cytosol and nucleus due to triggered release in early endosomes (~pH 6) and high cytotoxicity in Doxorubicin resistant MCF-7 (MCF-7/DOXR) cells due to internalization via folate-receptor mediated endocytosis to overcome P-gp (Lee et al., 2005). Folate functionalized micelles co-encapsulating Paclitaxel and Verapamil in O-carboxymethylated chitosan modified with deoxycholic acid revealed greater cytotoxicity and higher cellular uptake in drug resistance MCF-7 and multi-drug-resistant MCF-7/ADR cells through synergistic effect of folate receptor-mediated endocytosis and Verapamil mediated efflux mechanism to overcome drug resistance in tumor cells (Wang et al., 2011a). Wei et al. revealed that pluronics lowered the IC_50_ in human lung adenocarcinoma cell lines SPC-A1 (8.7 ± 0.4 ng/ml) and A-549 (0.10 ± 0.04 μg/ml) with Paclitaxel-Pluronic P123/F127 mixed polymeric micelles compared to Taxol and free Paclitaxel (Wei et al., 2009). Lu et al. developed dendrimer phthalocyanine-encapsulated polymeric micelle with Doxorubicin and revealed nuclear accumulation of Doxorubicin in doxorubicin-resistant MCF-7 breast cancer cells and xenograft model after photoirradiation with higher antitumor activity compared to photodynamic therapy alone (Lu et al., 2011). Cambón et al., synthesized reverse poly(butylene oxide)-poly(ethylene oxide)-poly(butylene oxide) block copolymers with potential P-gp inhibitory action in MDR cell line. Doxorubicin loaded in these polymeric micelles enhanced cell accumulation and cytotoxicity in MDR ovarian NCIADR-RES cell line over expressing P-gp (Cambón et al., 2013). Methotrexate conjugated mixed micelles of pluronic F127 and P105 suppressed tumor growth in KBv MDR cells compared to physically entraped mixed micelles due to combined effect of tumor chemosensitization by pluronic and passive targeting by micelles (Chen et al., 2013). Vincristine sulfate nanocarriers improved cellular uptake, cytotoxicity in MCF-7 and P-gp over expressing MCF-7/Adr resistant cancer cells by bypass P-gp due to endocytosis mediated by clathrin and caveolae pathways (Zhang et al., 2011b). Chemosensitizing ability of pluronics suppressed Doxorubicin induced MDR in murine lymphocytic leukemia cells (P388) and in BDF1 mice bearing (P388) ascite cancer cells with Doxorubicin-Pluronic P85 micelles (Sharma et al., 2008). Pluronics modulate MDR by intracellular ATP depletion, decreased mitochondrial potential and passive targeting. IC_50_ values of Paclitaxel-Pluronic P123/F127 mixed micelles revealed anti-proliferation activity against lung resistance protein over expressing human lung adenocarcinoma A-549 cells with 3-fold longer mean residence time and 31.8% reduction in tumor volume compared to Taxol after 28 days (Wei et al., 2010). Polyethylene glycol-polycaprolactone or Pluronic P105 micelle down-regulated the mitochondrial membrane potential and reduced ATP level to improve cytotoxicity (4 times), intracellular accumulation and overcome Doxorubicin resistance in human myelogenous leukemia (K562/ADR) cells compared to Doxorubicin solution at 12 ng/mL (Han et al., 2011).

### Mesoporous silica nanoparticles (MSNPs)

**Figure 2:**
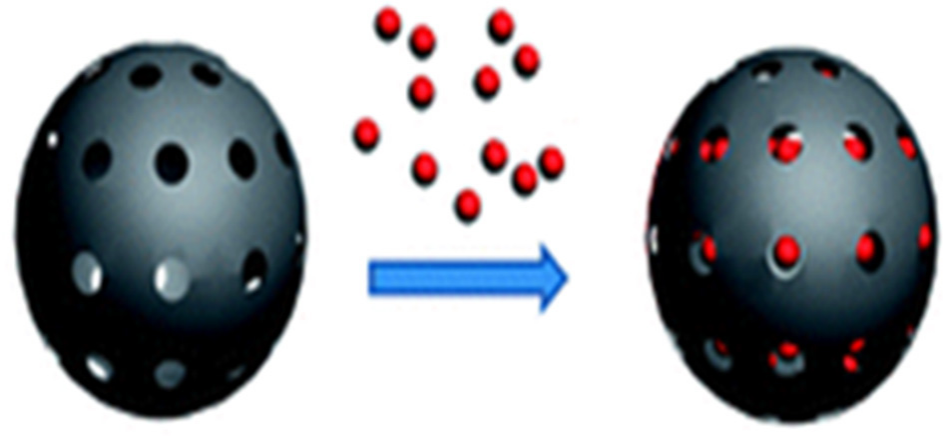
**Mesoporous silica nanoparticles (MSNPs)**.

Mesoporous silica nanoparticles (Figure 2) have high drug loading due to high pore volume and surface area, multifunctionalization for targeted and controlled delivery, enhanced cellular uptake and delivers therapeutics at cellular levels in cancer (Mai and Meng, 2013; Mamaeva et al., 2013). Doxorubicin MSNPs surface conjugated with TAT peptide facilitated intranuclear drug localization in multidrug resistant MCF-7/ADR cancer cells and overcome MDR compared to free Doxorubicin or non-TAT peptide conjugated nanoparticles (Pan et al., 2013). Doxorubicin MSNPs lowered the IC_50_ value 8-fold compared to free Doxorubicin and overcome MDR in Doxorubicin resistant and P-gp over expressing cancer cell line MCF-7/ADR by increased cell proliferation suppression effect (Shen et al., 2011). Chemotherapy efficacy was enhanced bypassing the efflux pump resistance in multidrug-resistance cancer cells by co-delivery of Doxorubicin and siRNAs in MSNPs (Chen et al., 2009a). Rapid internalization of siRNA loaded magnetic MSNPs coated with polyethylenimine and surface modified with fusogenic peptide (KALA) in the tumor cells resulted in knockdown of enhanced green fluorescent protein (EGFP) and VEGF and inhibited tumor growth by suppression of tumor neovascularization (Li et al., 2013b). Doxorubicin-CTAB micelles co-loaded pH responsive MSNPs overcome multi-drug resistance in both drug-resistant MCF-7/ADR cells and drug-sensitive MCF-7 cells due to chemosensitization potential of CTAB arresting the cell cycle and inducing apoptosis (He et al., 2011). Manganese oxide-based MSNPs loaded with Doxorubicin multifunctionalized as theranostics circumvented MDR, restored drugs anti-proliferative effect by endocytosis, P-gp inhibition and ATP depletion in cancer cells (Chen et al., 2012). Anticancer drug loaded magnetic MSNPs were internalized by A549 cells through an energy-dependent clathrin induced endocytosis pathway and inhibited cancer cell growth under magnetic field (Liu et al., 2012; Sekhon, 2012). Exposure of Doxorubicin loaded zinc doped iron oxide nanocrystals in mesoporous silica framework surface-modified with pseudorotaxanes to AC field caused death of (MDA-MB-231) breast cancer cells (Thomas et al., 2010). Hyperthermia stimulated the intracellular GSH level in A549 human lung cancer cells and enhanced anti-cancer efficacy of Doxorubicin MSNPs by inducing cell death and apoptosis (Lee et al., 2011). Lejiao Jia et al. developed Paclitaxel MSNPs and revealed that anti-tumor activity of Paclitaxel in breast cancer cells (MCF-7) was dependent on pore-size and apoptosis increased with increased nanoparticle pore size (Jia et al., 2013). Galactose functionalized Camptothecin MSNPs with photosensitizer (porphyrin) enhanced anti-cancer activity in human cell lines of colorectal (HCT-116), pancreatic (Capan-1) and breast cancer (MDA-MB-231) (Gary-Bobo et al., 2012).

### Other inorganic nanoparticles

Inorganic nanoparticles for cancer therapy include quantum dots, carbon nanotubes, silica nanoparticles, gold nanoparticles, iron oxide magnetic nanoparticles and ceramic nanoparticles. Doxorubicin covalently bounded to polyethylenimine via pH sensitive hydrazone linkage and conjugated to iron oxide nanoparticles functionalized with polyethylene glycol circumvented MDR and reduced cell viability in DOX-resistant cells over-expressed in rat glioma C6 cells compared to free drug (Kievit et al., 2011). Wu Yanan et al. studies reversed the effect of 5-Bromotetrandrine and magnetic iron oxide nanoparticle combining Daunorubicin in xenograft leukemia model and inhibited expression of Bcl-2 protein and up-regulated BAX and CASPASE-3 protein expression in K562/A02 cells xenograft tumor (Yanan et al., 2009). Doxorubicin loaded pH sensitive poly (beta-amino ester) copolymer superparamagnetic iron oxide nanoparticle in drug-resistant C6 glioma cell lines (C6-ADR) revealed 300% higher cellular internalization 24 h post-treatment and reduced IC_50_ by 65% at 72 h post-treatment compared to free Doxorubicin (Fang et al., 2012). Co-administration of Doxorubicin and magnetite nanoparticles in presence of magnetic field showed cytotoxic effects against breast cancer cell lines MDA-MB-468 with greater than 80% cell death in hyperthermia combination than with Doxorubicin alone (Sadeghi-Aliabadi et al., 2013). Similar drug resistance inhibitory effect of magnetite nanoparticles loaded with Doxorubicin and Tetrandrine against K562 leukemia cells have been reported by Wang et al. (2007), Chen et al. (2008). Significant reduction in transcriptions of Mdr-1 and Bcl-2 gene and increased expressions of Bax and caspase-3 in K562-n and K562-n/VCR cells *in-vivo* in nude mice revealed the potential of Daunorubicin magnetic nanoparticles to overcome multi-drug resistance (Chen et al., 2009b). MDR1-siRNA encapsulated magnetic chitosan iron oxide nanoparticle reversed MDR effect on MDR1 gene in BT325 glioblastoma cell line with 70–80% transfection efficiency by reduced expression of MDR1 at mRNA and protein level and decreased IC_50_ values in normal BT325 and transfected cell (Zhao et al., 2013). Lectin functionalized Paclitaxel magnetic nanoparticles lowered the IC_50_ with higher cellular uptake and cytotoxic effect on Bcr-Abl positive K562 cells in chronic myelogenous leukemia (Singh et al., 2011). Cisplatin magnetic nanoparticles enhanced inhibition of A549 cells and cisplatin-resistant A549 cells in MDR lung cancer cells, lowered the levels of MRP1, lung resistance-related protein, Akt and Bad pathways and increased the levels of caspase-3 genes and proteins (Li et al., 2013a). Single drug Tetrandrine loaded magnetic nanoparticles revealed a 100-fold lowering in mdrl mRNA level but no reduction in total P-gp content while magnetic nanoparticles loaded with Adriamycin and Tetrandrine synergistically reversed multidrug resistant in K562/A02 resistant cell lines (Chen et al., 2008). Heparin coated Doxorubicin super-paramagnetic iron oxide nanoparticles promoted apoptosis due to regulation of anti-apoptotic genes including caspase-3, bax, bcl-2 and surviving in human ovarian cancer cell lines A2780 (Javid et al., 2011).

Gold nanoparticles (AuNPs) are versatile platform for cancer drug delivery (Kumar and Liang, 2011; Kumar et al., 2013) and have recently entered cancer clinical trials phase I and II (Thakor et al., 2011; Vigderman and Zubarev, 2012). Gu et al. successfully synthesized doxorubicin grafted-PEGylated gold nanoparticles to overcome Doxorubicin resistant in cell lines (Gu et al., 2012). Oxaliplatin grafted on PEGylated AuNPs rapidly distributed in the nucleus and enhanced the chemotherapeutic efficacy (Brown et al., 2010). AuNPs surface conjugated with therapeutic peptide (PMI or p12) and targeted peptide (CRGDK) was rapidly internalized for better efficacy in overcoming breast cancer (Kumar et al., 2012). AuNPs covalently grafted with doxorubicin through thioctic acid-PEG linker inhibited growth of drug resistant breast cancer cells due to high drug concentrations inside cancer cells due to acid sensitive release from endosomes (Wang et al., 2011b). Zhang et al. observed similar effects with gold nanoparticle–DNA–paclitaxel conjugate (Zhang et al., 2011d). Gold nanorods functionalized with gastrin-releasing peptide (Bombesin) showed uptake via GRP receptor-mediated endocytosis with high binding affinity to breast cancer cells (Chanda et al., 2009, 2010). Selenium nanoparticles significantly enhanced the expression of pp38, Bax and cytochrome C in estrogen receptor-α positive cells (MCF-7) but not in estrogen receptor-α-negative cells (MDA-MB-231) and prevented mammary tumor growth by inducing cell death (Vekariya et al., 2012).

Quantum dots are semiconductor inorganic fluorescent nanocrystals with small and uniform sizes (1–20 nm), high surface to volume ratio, surface conjugation with multiple ligands and biocompatibility (Zhang et al., 2008a; Geszke-Moritz and Moritz, 2013). Water-soluble cadmium telluride (CdTe) quantum dots capped with negatively charged 3-mercapitalpropionic acid combined with Daunorubicin as a biomarker for simultaneous cellular imaging and inhibition of MDR for treatment of drug-sensitive leukemia K562 and drug-resistant leukemia K562/A02 cell lines was developed by Yanyan Zhou et al. The study revealed significant drug uptake in target cancer cells and cytotoxicity suppression in both cell lines (Zhou et al., 2010). Further Zhang et al. demonstrated rapid uptake and increased apoptosis rate which activated apoptosis-related caspases protein expression in drug-resistant human hepatoma HepG2/ADM cells with Daunorubicin-3-mercaptopropionic acid-capped Cadmium telluride quantum dots (Zhang et al., 2011a). Paclitaxel-loaded PLGA quantum dots were more cytotoxic than free Paclitaxel in paclitaxel-resistant KB paclitaxel-50 cells than paclitaxel-sensitive KB, however treatment with Verapamil reversed the MDR activity and reduced viability of KB paclitaxel-50 cell (Kuo et al., 2009). Doxorubicin conjugated via pH-sensitive hydrazone bond and aptamer to quantum dots when targeted to mutated MUC1 mucin over expressed in ovarian carcinoma revealed higher cytotoxicity than free drug with preferential accumulation in ovarian tumor and drug release in acidic environment of cancer cells (Savla et al., 2011).

### Dendrimers

Novel delivery systems comprising of Doxorubicin, dendrimer and vector protein rAFP3D to bind alpha-fetoprotein receptors on tumor cell surface accumulated in the cells by receptor mediated endocytosis and demonstrated high cytotoxicity against human ovarian adenocarcinoma cell lines - Doxorubicin-sensitive SKOV3 cells and Doxorubicin-resistant SKVLB cells revealed low toxicity against human peripheral blood lymphocytes reversing the MDR in Doxorubicin-resistant cells (Yabbarov et al., 2013). The cancer-targeting potential of folate/dextran/galactose ligands anchored on poly(propylene imine) dendrimers evaluated on HeLa and SiHa cell lines indicated an IC_50_ values of 0.05, 0.2, 0.8 and 0.08 μM for folate, dextran, galactose formulations and free paclitaxel respectively on HeLa cells while the IC_50_ values of 0.6, 0.8, 10 and 6 μM with folate, dextran and galactose formulations and free PTX respectively with SiHa cells. The study revealed the targeting potential of ligands in the order folate > dextran > galactose (Kesharwani et al., 2011). Dendrimer phthalocyanine-encapsulated polymeric micelle combined with doxorubicin and mediated by photochemical internalization showed doxorubicin release from endo-lysosomes to nuclei after photoirradiation and nuclear accumulation of doxorubicin, higher antitumor activity than DPc/m-PDT alone in drug-resistant MCF-7 cells and xenograft model (Lu et al., 2011). Biotin, a cell growth promoter is required for rapid proliferation of cancer cells and is over-expressed on cancer cell surface than normal tissue. Bifunctional dendrimer conjugated with biotin a targeting moiety and fluorescein isothiocyanate an imaging moiety exhibited higher cellular uptake by an energy-dependent process in HeLa cells than conjugate without biotin. Conjugation of targeting moieties such as sugar, folic acid, antibody, peptide and epidermal growth factor to dendrimers leads to preferential accumulation of drug in the targeted tissue or cells. Similar biotin-conjugate carriers have been reported to increase uptake of anti-cancer drugs in tumor cells (Yang et al., 2009a). Cytotoxicity of dendrimers-chlorambucil conjugate and inhibition of [3H] thymidine incorporated in DNA on both MDA-MB-231 and MCF-7 breast cancer cells demonstrated that the conjugate had more potent antiproliferative activity and actively inhibited collagen biosynthesis than chlorambucil (Bielawski et al., 2011). Similar cytotoxicity effects have been reported by Khandare et al. for conjugation of paclitaxel to linear PEG polymers and PAMAM dendrimers. PAMAM dendrimer-paclitaxel conjugate showed significantly higher toxicity while linear PEG-paclitaxel conjugate showed more than 25-fold lower toxicity compared to free drug with increased IC_50_ dose (Khandare et al., 2006). Surface modified G3 PAMAM dendrimers with permeation enhancing lauryl chains conjugated with Paclitaxel via glutaric anhydride linker revealed the potential to cross cellular barriers in cell monolayers indicated by increased apparent permeability coefficient and increased cytotoxicity in both human colon adenocarcinoma cell line (Caco-2) and primary cultured porcine brain endothelial cells (PBECs). The interactions of hydrophobic lauryl moieties of L6-G3-glu-pac dendrimer conjugate with plasma membrane revealed 12-fold greater permeability across both cell monolayers than free Paclitaxel (Teow et al., 2013). Dendrimer conjugated with methotrexate a dual-acting molecule showed cytotoxicity due to its potent inhibitory activity against dihydrofolate reductase and binds folic acid receptor, upregulated on cancer cell surface (Li et al., 2012b).

### Nanostructured lipid carriers (NLCs)

Mitoxantrone hydrochloride nanostructured lipid-dextran sulfate hybrid carriers enhanced cytotoxicity and invaded cells by clathrin-mediated endocytosis with high drug accumulation in BCRP overexpressing MCF-7/MX cells and overcome MDR compared to solution (Zhang et al., 2012). Oral bioavailability of Etoposide was enhanced 1.8-, 3.0-, and 3.5-fold in NLCs, PEG40-NLCs and DSPE-NLCs respectively compared to suspension. Etoposide DSPE-NLCs and NLCs revealed highest cytotoxicity, lower cellular viability and strong inhibitory effects against human epithelial-like lung carcinoma cells (A549) than etoposide with IC_50_ values of 40.61 ± 6.15 nM, 61.78 ± 7.49 nM, and 210.87 ± 0.76 nM respectively after 24 h (Némati et al., 1996; Zhang et al., 2011c). Oleh Taratula et al. developed dual targeting NLCs loaded with an anticancer drug (Doxorubicin or Paclitaxel) to induce cell death and siRNA to target MRP1 mRNA and BCL2 mRNA to suppress pump and nonpump cellular resistance in lung cancer cells respectively and overcome resistance. Further conjugation of targeting moiety Luteinizing Hormone Releasing Hormone (LHRH peptide) to NLCs enhanced the targeting specificity to cancer cells overexpressing LHRH receptors (Taratula et al., 2013). Folate decorated Paclitaxel and Doxorubicin loaded NLCs designed by Xing-Guo Zhang et al. exhibited high cytotoxicity against human breast cancer (MCF-7) cells and multi-drug resistant (MCF-7/ADR) cells with Paclitaxel NLCs and in MCF-7/ADR cells with Doxorubicin NLCs with MDR reversal potential of 34.3-fold for Paclitaxel NLCs and 6.4-fold for Doxorubicin NLCs. Similar cytotoxicity trend was observed against human ovarian cancer (SKOV3) cells and multi-drug resistant (SKOV3_TR_) cells with reversal power of 31.3 and 2.2-fold for Paclitaxel NLCs and Doxorubicin NLCs respectively compared to Taxol and Doxorubicin solution (Zhang et al., 2008b). Potential of active targeting the low density lipoprotein (LDL) receptors over expressed on cancer cells was utilized by Jaber Emami et al. and developed Paclitaxel loaded cholesterol NLCs which were taken by human colorectal cancer cell line (HT-29) through LDL receptor endocytic pathway and revealed IC_50_ values of 5.24 ± 0.96 ng/mL compared to 8.32 ± 1.35 ng/mL of free Paclitaxel solubilized in Cremophor-EL after 72 h exposure (Emami et al., 2012). Folate decorated Paclitaxel and Doxorubicin NLCs exhibited high cytotoxicity in MCF-7 and MCF-7/ADR cells while Doxorubicin NLCs revealed high cytotoxicity only in MCF-7/ADR cells compared to Taxol and Doxorubicin solution, while Paclitaxel and Doxorubicin NLCs revealed same cytotoxicity trends against human ovarian cancer cells (SKOV3) and their multidrug resistant (SKOV3_TR_) cells. The reversal power of Paclitaxel and Doxorubicin NLCs were 34.3- and 6.4-folds, respectively (Zhang et al., 2008b).

Nanovehicles enhance chemotherapeutics solubility, bioavailability, therapeutic index and overcome dose-limiting toxicity, non-specific biodistribution, non-targeting and emerging drug resistance in cancer therapy. Multifunctional nanocarriers along with distinct size and surface characteristics are able to target tumor cells through active and passive targeting approaches. Nanocarrier's ability to down regulate ABC transporters or carry gene expression modulator/inhibitor enhance drugs intracellular tumor concentrations improving the chemotherapeutic efficacy. Thus nanotechnology is a novel approach for specific delivery of chemotherapeutics with potential to overcome complexity of MDR in tumors treatments.

## Nanocarriers inhibiting MDR based on drug efflux pumps

### Silencing of drug resistance genes

RNAi technology has been explored as a therapeutic strategy to overcome MDR by silencing drug efflux transporter genes such as P-gp/MDR1 and MRP1. RNAi mediated silencing through siRNA, through transfection with shRNA (Saad et al., 2008; MacDiarmid et al., 2009; Chen et al., 2010; Patil et al., 2010) and decreased MDR1 expression with antisense oligodeoxynucleotides (Wang et al., 2010) are strategies to overcome P-gp associated MDR using RNAi. Targeting transferrin receptors with PEG coated siRNA nanoparticles silenced target gene M2 ribonucleotide reductase in refractory metastatic melanoma (Davis et al., 2010). Cationic and anionic liposome polycation-DNA nanoparticles loaded with C-Myc siRNA and Doxorubicin suppressed MDR1 gene expression via silencing the transcription level by targeting transcription factors, intercalation of Doxorubicin, topoisomerase II inhibition, transcription inhibition of resistant tumors and tumor regression (Chen et al., 2010). Sigma receptors overexpressed on non-small cell lung cancer, breast tumor and prostate cancer targeted with anisamide decorated nanoparticles reduced tumor growth of C-Myc siRNA, down-regulated MDR1 expression and increased Doxorubicin accumulation in xenograft model of NCI/ADR-RES (OVCAR-8 derived) tumor (Banerjee et al., 2004). Bacterially derived minicells encapsulating siRNA targeting MDR1 gene transcripts with cytotoxic drugs down-regulated P-gp and increased survival of mice bearing human tumor xenografts (MacDiarmid et al., 2009).

### Inhibition of drug resistance proteins

To overcome MDR, colloidal carries inhibiting drug resistance proteins P-gp includes polymeric nanoparticles (Khdair et al., 2009; Kuo et al., 2009; Patil et al., 2009a; Song et al., 2009), quantum dots (Kuo et al., 2009), liposomes (Wu et al., 2007), nanoemulsions (Ganta and Amiji, 2009) etc. which contains combination of P-gp inhibitors with anticancer drugs such as Paclitaxel, Vincristine, or Doxorubicin. Biotin or folic acid functionalized PLGA nanoparticles encapsulating Tariquidar and Paclitaxel resulted in higher cytotoxicity and inhibited tumor growth in human MDR tumor xenografts compared to Paclitaxel nanoparticles alone (Robey et al., 2008; Patil et al., 2009b). Paclitaxel loaded theragnostic PLGA nanoparticles conjugated to quantum dots were more effective than free Paclitaxel in Paclitaxel-sensitive nasopharyngeal KB carcinoma cells and Paclitaxel-resistant KB PTX-50 while cytotoxicity enhanced in presence of Paclitaxel-loaded nanoparticles with Verapamil (Kuo et al., 2009). Transferrin coated liposomes co-encapsulating Doxorubicin and Verapamil exhibited 5 and 3-fold cytotoxicity in Doxorubicin-resistant human erythroleukemia K562 cells compared to non-targeted liposomes and transferrin targeted liposomes with Doxorubicin alone respectively (Wu et al., 2007).

## Nanocarriers suppressing mechanism of drug resistance independent of efflux transporters

### Silencing of Bcl-2 and HIF1α gene expression

**Figure 3:**
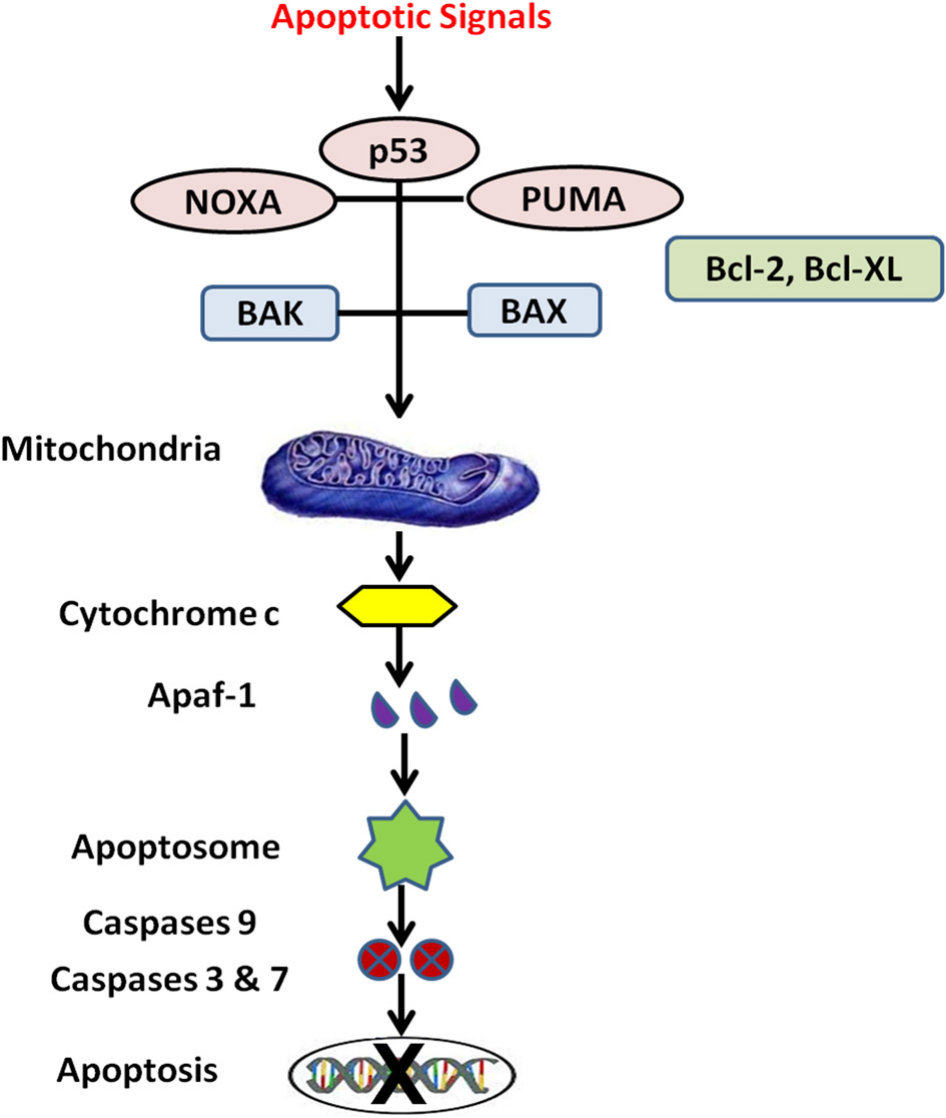
**Mitochondrial pathway**.

Nanotechnology approaches suppressing drug resistance mechanisms independent of drug efflux pumps are silencing of B-cell lymphoma 2 (Bcl-2) (Indran et al., 2011) (Figure 3) and hypoxia-inducible factor alpha (HIF1-α) genes. Bcl-2 family proteins are regulators of apoptosis and HIF1-α gene encodes a transcription factor in cellular response to hypoxia (Rapisarda and Melillo, 2009). Two isoforms of Bcl-2, Isoform 1 (1G5M) and Isoform 2 (1G5O/1GJH) exhibit similar fold antiapoptotic activity, however their ability to bind the BAD and BAK proteins suggest differences in antiapoptotic activity of the isoforms. Bcl-2 gene damage is a major cause of cancer and resistance to cancer treatments because over-expression of anti-apoptotic genes and under-expression of pro-apoptotic genes results in lack of cell death. Hypoxia regions present in solid tumors are indicators of malignant progression, metastatic development and chemoresistance. The degree of intra-tumoral hypoxia depends on expression of HIF-1 which is composed of 2 sub-units HIF-1α and HIF-1β and is major factor for cell survival in hypoxic environment (O'Donnell et al., 2006). Matrine (active component of Sophora flavescence dry roots) in human gastric cancer MKN45 tumor cells activates caspase-3, 7 and up-regulates pro-apoptotic molecules Bok, Bak, Bax, Puma, Bim and induces apoptosis via Bcl-2 (Noguchi et al., 2003; Luo et al., 2007). Cationic cholesterol derivative with hydroxyethylamino head group, cholesteryl-3bcarboxyamidoethylene-*N*-hydroxyethylamine (I) on liposome significantly promoted gene transfection, Bcl-2 antisense phosphorothioate oligonucleotides complexed with cationic liposomes suppressed human cancer cell growth and induced apoptosis in human cervix epithelial carcinoma cell lines HeLa and mouse fibroblast NIH3T3 cells (Okayama et al., 1997). Positively charged chitosan coated PLGA nanoparticles with siRNA increased transfection and blocked the expression of anti-apoptotic Bcl-2 gene with significant cellular uptake and tumor regression (Jagani et al., 2013). Dong-feng Yu et al. developed cationic liposomes to downregulate the expression of Bcl-2 gene with siRNA transfection with enhanced apoptosis and sensitivity of 5-Fluorouracil in gastric adenocarcinoma SGC-7901 cell (Yu et al., 2013). Suppression of Bcl-xL gene through co-delivery of Doxorubicin and small hairpin RNA (shRNA) in polyplexes conjugated with an anti-PSMA aptamer specifically binds the prostate-specific membrane antigen expressed on prostate cancer cell surface. Aptamer polyplexes revealed excellent tumoricidal efficacy and significantly lowered the IC_50_ values by 17-fold compared to mixture of shRNA and Doxorubin (Kim et al., 2010a). Gene silencing capability of siRNA loaded magnetic MSNPs coated with polyethyleneimine effectively knock downed both exogenous enhanced green fluorescent protein (EGFP) gene and endogenous Bcl-2 gene with negligible cytotoxicity and released siRNA in cancer cells (Li et al., 2011). Glycoprotein transferrin (Tf) is a ligand for transferrin receptors (TfR) overexpressed on cancer cells and internalized by receptor-mediated endocytosis. Novel transferrin receptor-targeted liposomes delivered phosphorothioate antisense oligodeoxyribonucleotide (ODN-G3139) in TfR positive K562 leukemia cells and downregulated Bcl-2 protein in K562 cells 2-fold greater than non-targeted liposomes and 10-fold greater than free G3139. Tf-conjugated liposomes with G3139 reduced Bcl-2 transcription by >80%, lowered IC_50_ from 1.8 to 0.18 μM and sensitized K562 cells to Daunorubicin (Chiu et al., 2006).

### Modulation of ceramide levels

Ceramide lipids are endogenous lipids and potent mediators of cellular responses in cancer including apoptosis, cell growth suppressor, differentiation, cell migration and adhesion. Ceramides are located in cell membranes and mitochondrial outer membrane, releasing pro-apoptotic factors Cytochrome c by forming permeable channels. Few sphingolipids are vital signal transducer and cell regulator in growth suppression and apoptosis. Extracellular agent such as tumor necrosis factor α activates sphingomyelinase and cleaves membrane sphingomyelin to generate cellular ceramide. Ceramide are converted to sphingolipids in presence of P-gp and accelerates cancer cell death by co-administration of P-gp antagonists with short-chain ceramides (C_6_-ceramide) (Hannun and Obeid, 1995; Pettus et al., 2002; Boddapati et al., 2008). Exposure to chemotherapy and/or anticancer drugs increase intracellular ceramide levels in cancer cells and is involved in membrane clustering of the death receptor. Most anticancer chemotherapeutics stimulate ceramide accumulation through increased ceramide synthesis or inhibition of ceramide catabolism. Neutralization of ceramide via glycosylation or phosphorylation in malignant cells is linked to MDR. New therapeutic strategies to overcome resistance focus on increasing endogenous ceramide levels by stimulating ceramide synthesis, inhibiting ceramide neutralization, or direct delivery of exogenous ceramide (Barth et al., 2011). Cytotoxicity of C_6_-ceramide nanoliposomes with P-gp antagonist (Tamoxifen, Cyclosporine-A, VX-710 (Biricodar), Verapamil) in human CRC cell lines (HCT-15, HT-29, LoVo) revealed synergistic effect of caspase dependent apoptosis, poly ADP ribose polymerase(PARP) cleavage, DNA fragmentation, cell cycle arrest, increased mitochondrial membrane permeability and enhanced protein expression of tumor suppressor p53 (Morad et al., 2013). Shabbits and Mayer (2003) revealed that cytotoxicity and cellular uptake of ceramides are dependent on acyl chain length with the most active C_6_-ceramide (IC_50_ value = 3–14 μM) and least active C16-ceramide (IC_50_ value = 100 μM) in MDA435/LCC6 human breast cancer and J774 mouse macrophage cell lines (Shabbits and Mayer, 2003). Cisplatin-Fe_3_O_4_ magnetic nanoparticles reversed resistance of ovarian carcinoma cell line SKOV3/DDP by 2.2-fold and down-regulated mRNA levels of Bcl-2 and survivin expression with increased cell apoptosis (Jiang et al., 2009). Similarly Daunorubicin-Fe_3_O_4_ magnetic nanoparticles lowered the transcriptions of Mdr-1 and Bcl-2 gene and increased the transcriptions and expressions of Bax and caspase-3 in K562-n and K562-n/VCR cells in nude mice to overcome MDR (Chen et al., 2009b). Lonidamine and Paclitaxel dual loaded PLGA/PEG/EGFR-peptide targeted nanoparticles at 1 μM paclitaxel/10 μM lonidamine dose revealed <10% cell viability for all hypoxic cell lines and <5% cell viability for all normoxic cell lines overexpressing EGFR in human breast and ovarian cancer cell lines. EGFR-peptide targeted nanoparticles promoted mitochondrial binding of Bcl-2 proteins (Lonidamine) and hyperstabilizing microtubules (Paclitaxel) to overcome MDR (Milane et al., 2011). siRNA cationic polymeric nanoparticles downregulated Bcl-2 mRNA expression levels (<10%) in HepG2, HeLa and MDA-MB-231 cell lines and sensitized HeLa cells to Paclitaxel (Beh et al., 2009). Transferrin targeted protamine lipid nanoparticles of antisense oligonucleotide (G3139) down-regulated Bcl-2 to overcome resistance in K562, MV4-11 and Raji leukemia cell lines and was more effective than non-targeted lipid nanoparticles and frees G3139 and induced caspase-dependent apoptosis (Yang et al., 2009b). Co-administration of Paclitaxel (20 mg/kg) and C_6_-ceramide (100 mg/kg) in poly(ethylene oxide)-modified poly(epsilon-caprolactone) nanoparticles revealed > 4.3- and 3-fold increase in tumor growth delay and 3.6- and 3-fold increase in tumor volume doubling time in wild-type SKOV-3 and multidrug resistant (MDR-1 positive) SKOV-3_TR_ models respectively compared to individual agents (Devalapally et al., 2007). Tumor accumulation of Paclitaxel from Paclitaxel-C_6_-ceramide poly (beta-amino ester) nanoparticles was high compared to free drug in sensitive MCF-7 and multidrug resistant MCF-7TR (MDR-1 positive) human breast adenocarcinoma (van Vlerken et al., 2008). C_6_-ceramide nanoliposomes revealed caspase-dependent apoptosis and diminished survivin protein expression in treatment of human and rat natural killer-large granular lymphocytic leukemia cells (Liu et al., 2010). C_6_-ceramide loaded temperature-sensitive linear-dendritic nanoparticle revealed preferential uptake of fluorescein isothiocyanate-labeled linear-dendritic nanoparticles into human MDA-MB-231 breast adenocarcinoma cells with growth inhibition and solid tumor apoptosis with hyperthermia (Stover et al., 2008). Cytotoxicity of cetyltrimethyl ammonium bromide stabilized SLNs loaded MBO-asGCS oligonucleotide with or without C_6_-ceramide evaluated in NCI/ADR-RES human ovary cancer cells revealed enhanced uptake of MBO-asGCS oligonucleotide with downregulation of GCS reversing resistance of cells to Doxorubicin (Siddiqui et al., 2010). Docetaxel loaded hyaluronic acid-ceramide nanoparticles enhanced intracellular uptake in CD44-overexpressing cell line (MCF-7) and revealed MDR effect in MCF-7/ADR cells (Cho et al., 2011). Doxorubicin loaded polyethylene glycol conjugated hyaluronic acid-ceramide revealed greater uptake in CD44 receptor expressed in SCC7 cell line (Cho et al., 2012). C_6_-ceramide nanoliposomal with Gemcitabine or an inhibitor of glucosylceramide synthase [D-threo-1-phenyl-2-decanoylamino-3-morpholino-1-propanol (PDMP)] in Gemcitabine resistant human pancreatic cancer cell line revealed cytotoxicity and inhibited tumor growth (Jiang et al., 2011). Transferrin modified ceramide liposomes initiated lysosomal membrane permeabilization resulting in leakage of hydrolytic enzymes (cathepsins) into cytoplasm, induced cancer cells apoptosis and revealed antitumor and pro-apoptotic effects in A2780-ovarian carcinoma xenograft mouse model compared to ceramide-free and ceramide-loaded non-modified liposomes (Koshkaryev et al., 2012). Co-administration of Tamoxifen and Paclitaxel in poly (ethylene oxide) modified poly (epsilon-caprolactone) polymeric nanoparticles enhanced antitumor efficacy, lowered IC_50_ of Paclitaxel by 10- and 3-fold in SKOV3 cells and P-gp over-expressing SKOV3_TR_ cells respectively (Devalapally et al., 2008). Polymeric nanoparticles co-encapsulating Paclitaxel and C_6_-ceramide enhanced apoptotic signaling and reduced tumor volume 2-fold over standard Paclitaxel monotherapy (van Vlerken et al., 2010). Ceramide-generating properties of 4-HPR (Fenretinide) are being evaluated in phase II study of recurrent ovarian cancer and C_6_-ceramide nanoliposomes are being evaluated as neoplastic-selective agent (Chapman et al., 2010).

### Targeting NF-κB

Transcriptional factor nuclear factor-kappa B (NF-κB) plays vital role in cancer development and resistance. Degradation of inhibitor κB after phosphorylation by inhibitor κB kinases activates NF-κB translocating into nucleus and initiating transcription contributing to tumor development, progression, chemoresistance, inflammation, and autoimmune diseases (Zingarelli et al., 2003; Li and Sethi, 2010). NF-κB is involved in multiple cellular processes including stress, cytokine gene expression, free radicals, cellular adhesion, cell cycle activation, apoptosis, and oncogenesis (Baud and Karin, 2009). NF-κB is activated via two distinct signal transduction pathways in cancer, the canonical and non-canonical pathways. NF-κB regulates expression of key proteins such as Bcl-2, Bcl-XL, cellular inhibitors of apoptosis, survivin, TRAF, Cox-2, MMP9, iNOS, and cell cycle regulatory components. Thus, NF-κB is a potential target for cancer therapeutics since inhibitors of NF-κB mediates antitumor responses and enhances tumor sensitivity to anticancer drugs (Luqman and Pezzuto, 2010). Activation of NF-κB affects cancer cell survival while inhibition of NF-κB enhances sensitivity of cancer cells to antineoplastic agents (Schwartz et al., 1999). NF-κB is important in tumorigenic process due to its strong anti-apoptotic functions in cancer cells (Magné et al., 2006). Polyethylene glycol-5000 coated Curcumin PLGA nanoparticles induced apoptosis of leukemic cells, inhibited TNF-induced NF-κB activation and suppressed NF-κB-regulated proteins involved in cell proliferation (cyclin D1), invasion (MMP-9) and angiogenesis (VEGF) (Nair et al., 2010). Micellar aggregates of cross-linked copolymers N-isopropylacrylamide with N-vinyl-2-pyrrolidone and poly(ethyleneglycol) monoacrylate encapsulating Curcumin induced cellular apoptosis, blocked NF-κB activation and down-regulated proinflammatory cytokines (IL-6, IL-8 and TNFα) in human pancreatic cancer cell lines (Bisht et al., 2007). Silica nanoparticles (50–200 μg/mL) generated reactive oxygen species, mitochondrial depolarization and apoptosis in human umbilical vein endothelial cells (HUVECs), activated c-Jun N-terminal kinase (JNK), c-Jun, p53, caspase-3 and NF-κB, increased Bax expression and suppressed Bcl-2 protein while the highest concentration significantly increased the necrotic rate, LDH leakage, expression of CD54 and CD62E and release of TF, IL-6, IL-8 and MCP-1 (Liu and Sun, 2010). Potential of gene therapy for targeting NF-κB has recently been explored as a new strategy in cancer (Tas et al., 2009). Degradation of TSP [Tween 85-s-s-polyethyleneimine (TSP)] a non-viral gene vector for p65 (shRNA) from TSP/p65 shRNA nanoparticles with release of shRNA blocked NF-κB signaling pathway, induced cell apoptosis and down-regulated p65 expression in breast cancer cells (Xiao et al., 2013). Inhibition of NF-κB with pyrrolidine dithiocarbamate (PDTC) an antioxidant and heavy metals chelator suppressed release of IκBα from NF-κB and induced cell death in neuroblastoma cells (Schreck et al., 1992). Doxorubicin and NF-κB inhibitor PDTC entrapped folic acid conjugated chitosan nanoparticles enhanced intracellular targeting of tumor cells via folic acid receptor mediated endocytosis and lowered IC_50_ values compared to free drug to overcome resistance (Fan et al., 2010).

## Nanocarriers addressing efflux pump dependent and independent drug resistance mechanisms

Simultaneously delivery of single/multiple anticancer agents in nanocarriers addressing both efflux pump dependent (P-gp) and independent (NF-κB) drug resistance mechanisms enhances cell apoptosis and induce cancer cell death. Paclitaxel and Curcumin (NF-κB and P-gp inhibitor) co-encapsulated in flaxseed oil nanoemulsion enhanced cancer cell sensitivity to Paclitaxel and cytotoxicity in SKOV3 and drug resistant SKOV3_TR_ human ovarian adenocarcinoma cells (Ganta and Amiji, 2009). Doxorubicin and siRNA containing cationic liposomes simultaneously silenced MRP1 and Bcl-2 (Saad et al., 2008). Doxorubicin/Mitomycin/5-Fluorouracil loaded hydroxyapatite nanoparticles acted synergistically with recombinant mutant human tumor necrosis factor-α (rmhTNFα) reduced P-gp levels of mRNA, increased intracellular concentration in human hepatoma xenografts of HepG2/ADM cells and suppressed tumor cell growth by apoptosis (Al-Bataineh et al., 2010; Ronaldson et al., 2010).

## Physical approaches to overcome MDR

### Drug delivery with thermal therapy

In hyperthermia therapy, cells undergo heat stress (41–46°C) resulting in activation and/or initiation of intracellular and extracellular degradation mechanisms like protein denaturation, protein folding, aggregation and DNA cross linking, changing tumor cell physiology and leading to apoptosis or making cancer cells more sensitive to anti-cancer drugs. Hyperthermia increases blood flow to the tumor cells and enhances delivery of nanocarriers and thus used as an adjunct treatment to increase efficacy of chemotherapy and enhance radiation induced tumor damage. Depending on the degree of temperature, hyperthermia is classified (i) in-thermo ablation; tumor subjected to >46°C (upto 56°C) causes cells to undergo direct tissue necrosis, coagulation or carbonization (ii) moderate hyperthermia (41–46°C) affects both cellular and tissue (iii) diathermia (<41°C) for rheumatic diseases. Cellular effects of moderate hyperthermia include induction and regulation of apoptosis, signal transduction and MDR. Super-paramagnetic iron oxide particles induced therapeutic hyperthermia; liposomal nanocarrier revealed high intra-tumoral accumulation of magnetic particles on application of magnetic field (100–120 kHz) to attain temperatures 40–45°C. Folate receptor targeted Doxorubicin liposomes with hyperthermia reduced IC_50_ in cervical carcinoma cells. Temperature sensitive poly(N-isopropylacrylamide) nanocarriers release anticancer drugs in presence of specific temperature triggers. Hyperthermia enables magnetic nanoparticles to enter tumor cells by generating heat in tissues/cells and is utilized for selective targeting through cancer-specific binding agents and controlled drug delivery over conventional hyperthermia (Chicheł et al., 2007; Kumar and Faruq, 2011).

### Drug delivery with ultrasound therapy

Ultrasound induces thermal effects and helps nanocarrier's extravasation in tumor, enhance drug diffusion through tumor interstitium, release drug from nanocarriers within tumor and increase intracellular drug accumulation on irradiation to enhance treatment of MDR cancer. Howard et al. demonstrated that sonication enhanced uptake of Paclitaxel 20-fold from micellar system in breast cancer tumor cell line and inhibited 90% cell proliferation. Doxorubicin-pluronic^®^ P105 micelles with ultrasound resulted in high intracellular drug accumulation in promyelocytic leukemia HL-60 cells, ovarian carcinoma drug sensitive and multidrug resistant cells (A2780 and A2780/ADR) and breast cancer (MCF-7) cells (Marin et al., 2002). Paclitaxel micelles of methoxy poly (ethylene glycol)-block-poly (D, L-lactide) enhanced intracellular drug accumulation 2-fold and cytotoxicity in drug-sensitive (MDCKII and MCF-7) and P-gp expressing (MDCKII-MDR and NCI-ADR) cell lines with ultrasound (Wan et al., 2012). Pure and mixed micelles of pluronic^®^ P105, PEG2000-diacylphospholipid and poly (ethylene glycol)-co-poly(β-benzyl-l-aspartate) loaded Doxorubicin with ultrasound treatment enhanced intracellular drug accumulation in ovarian carcinoma tumor model in nu/nu mice and inhibited tumor growth rate (Gao et al., 2005). Ultrasound therapy downregulated levels of P-gp, MRP and lung resistance protein to 62.84 ± 3.42%, 10.26 ± 1.18%, and 3.05 ± 0.37% in HepG2/ADM cells from 96.97 ± 2.41%, 20.84 ± 3.12%, and 1.16 ± 0.59% levels, respectively. Ultrasound increased percent Bax in HepG2/ADM cells leading to cellular apoptosis and MDR reversal (Liu et al., 2001; Rapoport, 2004; Howard et al., 2006; Kedar et al., 2010; Milane et al., 2011; Wu et al., 2011; Gao et al., 2012). The studies indicate potential of ultrasound waves to disrupt nanocarrier core, form micropores in cell membrane allowing diffusion of drugs and modulate membrane drug efflux pumps function. However which mechanisms of ultrasound (heat, cavitation or microstreaming) are predominantly involved in modulating drug efflux transporter on cell membrane is still unclear. The studies on these aspects are underway in our lab.

### Drug delivery with photodynamic therapy

Photodynamic therapy (PDT) has wide applications in cancer therapy due to its specificity and selectivity. PDT involves three components light, oxygen, and a photosensitizer (non-toxic drug) to achieve photocytotoxicity. PDT includes administration of a photosensitizer which specifically accumulates in cancer cells and when illuminated with red visible light (620–690 nm) generates reactive oxygen species in presence of tissue oxygen and causes cell death. Photofrin-2 (hematoporphyrin derivative) is the only PDT drug approved for clinical application in treatment of bladder, lung and esophageal cancer. Folic acid coated phospholipid-capped protoporphyrin IX (PpIX) loaded FITC-sensitized mesoporous silica nanocarriers (NanoPDT) effectively targeted receptors overexpressed on HeLa cells with high intracellular PpIX concentrations compared to free PpIX. NanoPDT on irradiation generated oxygen species, enhanced photocytotoxicity and inhibited 65% tumor growth in nude mice inoculated with B16F10 melanoma (Teng et al., 2013). Dendrimer phthalocyanine encapsulated polymeric micelle showed higher PDT efficacy in mice bearing human lung adenocarcinoma A549 cells, enhanced photocytotoxicity upon photoirradiation and accumulated in endolysosomes than Photofrin-2 (Nishiyama et al., 2009). However, polyethylene-glycol(PEG)-grafted transferrin-conjugated liposomes of second-generation photosensitizer 5, 10, 15, 20-tetra (*m*-hydroxyphenyl) chlorin (Foscan) did not improved the photocytotoxicity or intracellular accumulation of Foscan in esophageal cancer cell line when compared to unmodified liposomes (Paszko et al., 2013). Chitosan functionalized pluronic nanogel containing gold nanorod as photothermal therapy agent and chlorine e6 (Ce6) as photosensitizer for PDT enhanced tumor ablation *in-vivo* by combination of PDT followed by photothermal therapy compared to single therapy (Kim et al., 2013). Co-delivery of Docetaxel and photosensitizer zinc-phthalocyanine (ZnPc) loaded nanoparticles on irradiation decreased viability in HeLa cells after 72 h and enhanced antitumor activity in orthotopic amelanotic melanoma animal model compared to Docetaxel nanoparticles alone (Conte et al., 2013). Hyperbranched poly(ether-ester) (HPEE) loaded photosensitizer chlorin (e6) nanoparticles revealed better up taken by human oral tongue cancer CAL-27 cells after 4 h with cytoplasmic localization and higher phototoxicity compared to free ce6 after irradiation (Li et al., 2012c). Efficiency of second generation photosensitizers [2, 9, 17, 23-tetrakis-(1,6-hexanedithiol) phthalocyaninato] zinc (II) in PDT either free or encapsulated in gold nanoparticles/liposomes on photodamage of fibroblast and breast cancer cells revealed breast cancer cell damage with phthalocyanine liposomes while gold nanoparticles improved the effect with PDT (Nombona et al., 2012). Hematoporphyrin loaded liposomes revealed higher intratumoral accumulation of photosensitizer compared to free drug in MS-2 fibrosarcoma mouse model. Necrosis or apoptosis contributes to PDT mediated cell death in cancer and is due to generation of reactive oxygen species causing oxidative damage of cellular organelles. Thus, drug delivery in combination with PDT is a novel technique to selectively target cancer cells leading to cell lysis and overcome MDR in cancer therapy improving the efficacy.

## Nanotheranostics

**Figure 4:**
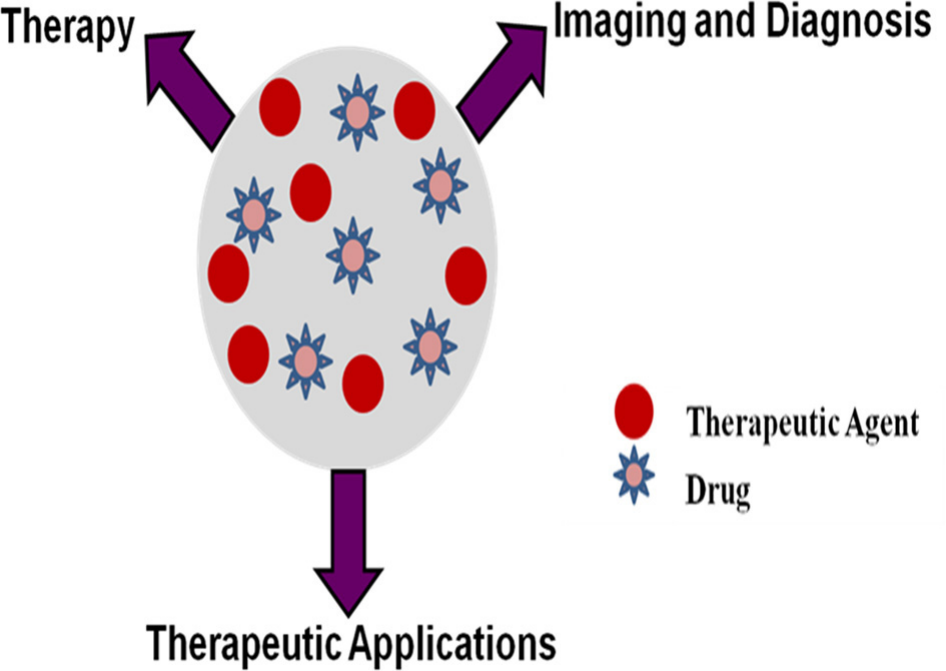
**Theragnostic agent**.

Nanotheranostics or quadrugnostic are third generation integrated nanovehicles comprising of four elements for diagnosis of tumor location/s, specific targeting to cancer cells, eradication of malignant cells with cytotoxic drug/s and neutralize drug resistance mechanism (Figure 4). Nanotheranostics serve dual roles as diagnostics and therapeutics thereby reducing chemotherapy dose, toxicity and side-effects to healthy tissues and increase therapeutic index (Ahmed et al., 2012). Superparamagnetic iron-oxide nanoparticles (SPIONs) with magnetic properties have excellent potential in tumor-targeting, diagnosis, monitoring and therapy (Santhosh and Ulrih, 2013). Polysorbate 80 coated Temozolomide-loaded PLGA superparamagnetic nanoparticles revealed higher intracellular uptake with antiproliferative effect on malignant brain glioma C6 cells compared to non-polysorbate 80 coated nanoparticles (Ling et al., 2012). Diagnostic, targeting and therapeutic potential of Doxorubicin loaded SPION and plasmonic gold nanoparticles have been evaluated in cancer treatment (Maeng et al., 2010). PEG and PEI-coated SPIONs increased intracellular concentration of Doxorubicin in resistant rat glioma C6-ADR cell line with IC_50_ values 3–5-fold lower compared to free Doxorubicin (Kievit et al., 2011). Fang et al. (2012) reported 3-fold lower IC_50_ with PBAE-coated SPIONs than free Doxorubicin in C6-ADR cells in accordance with Kievit et al. Gu et al. revealed that Doxorubicin loaded PEGylated gold nanoparticles were more cytotoxic on MDR cells compared to free Doxorubicin but less effective on sensitive cell lines (human hepatoma cells HepG2 and HepG2-R-a MDR subline). PEGylated Doxorubicin gold nanoparticles increased intracellular uptake and nuclear localization significantly up to 6 h by endocytosis (Gu et al., 2012). Theragnostic nanoparticles incorporating Doxorubicin or Paclitaxel for imaging and targeted chemotherapy were developed by Ahmed et al. (2012) and Kelkar and Reineke (2011). Paclitaxel loaded chitosan nanoparticles labeled with Cy5.5 (NIR fluorescence dye) were developed for imaging and cancer therapy in SCC7 tumor-bearing mouse models (Min et al., 2008; Kim et al., 2010b; Na et al., 2011; Ryu et al., 2011). Fluorescence property of Doxorubicin and photoluminescence of gold was utilized for imaging, monitoring drug uptake and tumor cells localization with folic acid decorated Doxorubicin gold nanorods (Newell et al., 2012). Chen et al. developed Doxorubicin encapsulated pH-responsive theragnostic nanoparticles with Cy5 for tissue targeting and imaging. Folate coated Doxorubicin SPIONs made of poly(ethylene oxide)-trimellitic anhydride chloride-folate increased anticancer efficacy in liver cancer, lowered expression of CD34 and Ki-67 markers of angiogenesis and cell proliferation respectively with 2- and 4-fold decrease in tumor volume compared to free Doxorubicin and Doxil^®^ respectively (Maeng et al., 2010). Acetylated dendrimer-entrapped gold nanoparticles were taken by cell lysosomes and detected under X-ray after incubation *in-vitro* and in xenograft tumor model after intratumoral and intraperitoneal administration for imaging human lung adenocarcinoma cell line (SPC-A1 cell) (Wang et al., 2011b). Cetuximab conjugated magneto-fluorescent silica nanoparticles for targeting EGFR receptor and *in-vivo* colon cancer imaging revealed high tumor uptake with application of an external magnetic field and MRI signal changes in human colon cancer xenograft mouse model (Cho et al., 2010). Folic acid-conjugated PEG-SPIONs labeled with Cy5.5 for imaging and active targeting to lung cancer resulted in higher intracellular uptake in KB cells and lung cancer model compared to non-folic acid coated nanoparticles (Yoo et al., 2012). Butyl rhodamine B fluorescent nanoparticles conjugated with anti-Her-2 monoclonal antibody were developed successfully for imaging and targeting ovarian cancer (Hun et al., 2008). Paclitaxel SPIONs significantly increased intracellular uptake and induced regrowth delay *in-vivo* in CT-26 cells with no toxicity (Schleich et al., 2013). Folic acid decorated Tamoxifen magnetic nanoparticles were developed for imaging and detection of human breast cancer cells that over express folic acid receptors (Majd et al., 2013). Immuno-targeted gold-iron oxide nanoparticles selectively accumulated in SW1222 xenograft colorectal tumors compared to non-antigen-expressing tumor xenografts. Photothermal treatment with IR irradiation revealed >65% of antigen-expressing tumor cells presented corrupt extracellular matrix and cytoplasmic acidophilia suggesting effectiveness of nanoparticle-assisted thermal therapy (Xie et al., 2010; Lu et al., 2012; Luk et al., 2012; Kirui et al., 2013).

## Conclusion

Nanodrug delivery systems are versatile platform for delivery of anticancer drugs and have been utilized successfully toward overcoming cancer drug resistance mechanisms, maximizing chemotherapeutic efficacy. Nanocarriers have effectively overcome challenges of limited aqueous solubility, low bioavailability, lack of targeting cancer tissues, increase drug therapeutic index, preferential accumulation by EPR effect and divert ABC-transporter mediated drug efflux MDR with potential to be multi-functionalized for cancer treatment. Nanocarriers promise to alleviate many challenges in clinical cancer therapy to benefit patients in future. Cancer science is progressing rapidly and understanding the molecular basis of drug resistance in cancer promises more effective treatments.

### Conflict of interest statement

The authors declare that the research was conducted in the absence of any commercial or financial relationships that could be construed as a potential conflict of interest.

## References

[B1] Ahmed N. Fessi H. Elaissari A. (2012). Theranostic applications of nanoparticles in cancer. Drug Discov. Today 17, 928–934 10.1016/j.drudis.2012.03.010 22484464

[B2] Al-Bataineh M. van der Merwe D. Schultz B. Gehring R. (2010). Tumor necrosis factor alpha increases P-glycoprotein expression in a BME-UV *in vitro* model of mammary epithelial cells. Biopharm. Drug Dispos. 31, 506–515 10.1002/bdd.731 21104926 PMC3034978

[B3] Alberto G. Hilary S. Aviva T. Samuel Z. Wang S. Lee R. (2004). Tumor cell targeting of liposome entrapped drugs with phospholipid-anchored folic acid–PEG conjugates. Adv. Drug Deliv. Rev. 56, 1177–1192 10.1016/j.addr.2004.01.011 15094214

[B4] Antony A. (1996). Folate receptors. Annu. Rev. Nutr. 16, 501–521 8839936 10.1146/annurev.nu.16.070196.002441

[B5] Ayers D. Nasti A. (2012). Utilisation of nanoparticle technology in cancer chemoresistance. J. Drug Deliv. 2012:265691 10.1155/2012/265691 23213536 PMC3505656

[B6] Banerjee R. Tyagi P. Li S. Huang L. (2004). Anisamide-targeted stealth liposomes: a potent carrier for targeting doxorubicin to human prostate cancer cells. Int. J. Cancer 112, 693–700 10.1002/ijc.20452 15382053

[B7] Barth M. Brian C. Cabot M. Kester M. (2011). Ceramide-based therapeutics for the treatment of cancer. Anticancer Agents Med. Chem. 11, 911–919 10.2174/187152011797655177 21707481

[B8] Baud V. Karin M. (2009). Is NF-κB a good target for cancer therapy? Hopes and pitfalls. Nat. Rev. Drug Discov. 8, 33–40 10.1038/nrd2781 19116625 PMC2729321

[B9] Beh C. Seow W. Wang Y. Zhang Y. Ong Z. Ee P. (2009). Efficient delivery of Bcl-2-targeted siRNA using cationic polymer nanoparticles: downregulating mRNA expression level and sensitizing cancer cells to anticancer drug. Biomacromolecules 10, 41–48 10.1021/bm801109g 19072631

[B10] Bernardi A. Braganhol E. Jäger E. Figueiró F. Edelweiss M. Pohlmann A. (2009). Indomethacin loaded nanocapsules treatment reduces *in-vivo* glioblastoma growth in a rat glioma model. Cancer Lett. 281, 53–63 10.1016/j.canlet.2009.02.018 19286307

[B11] Bielawski K. Bielawska A. Muszyńska A. Popławska B. Czarnomysy R. (2011). Cytotoxic activity of G3 PAMAM-NH_2_ dendrimer-chlorambucil conjugate in human breast cancer cells. Environ. Toxicol. Pharmacol. 32, 364–372 10.1016/j.etap.2011.08.002 22004955

[B12] Bisht S. Feldmann G. Soni S. Ravi R. Karikar C. Maitra A. (2007). Polymeric nanoparticle-encapsulated Curcumin (“nanocurcumin”): a novel strategy for human cancer therapy. J. Nanobiotechnol. 5:3 10.1186/1477-3155-5-3 17439648 PMC1868037

[B13] Boddapati S. D'Souza G. Erdogan S. Torchilin V. Weissig V. (2008). Organelle-targeted nanocarriers: specific delivery of liposomal ceramide to mitochondria enhances its cytotoxicity *in vitro* and *in vivo*. Nano Lett. 8, 2559–2563 10.1021/nl801908y 18611058

[B14] Brown S. Nativo P. Smith J. Stirling D. Edwards P. Venugopal B. (2010). Gold nanoparticles for the improved anticancer drug delivery of the active component of oxaliplatin. J. Am. Chem. Soc. 132, 4678–4684 10.1021/ja908117a 20225865 PMC3662397

[B15] Broxterman H. Lankelma J. Hoekman K. (2003). Resistance to cytotoxic and anti-angiogenic anticancer agents: similarities and differences. Drug Resist. Updat. 6, 111–127 10.1016/S1368-7646(03)00026-8 12860459

[B16] Cambón A. Rey-Rico A. Mistry D. Brea J. Loza M. Attwood D. (2013). Doxorubicin-loaded micelles of reverse poly(butylene oxide)-poly(ethylene oxide)–poly(butylene oxide) block copolymers as efficient “active” chemotherapeutic agents. Int. J. Pharm. 445, 47–57 10.1016/j.ijpharm.2013.01.056 23380628

[B17] Chanda N. Kattumuri V. Shukla R. Zambre A. Katti K. Upendran A. (2010). Bombesin functionalized gold nanoparticles show *in-vitro* and *in-vivo* cancer receptor specificity. Proc. Natl. Acad. Sci. U.S.A. 107, 8760–8765 10.1073/pnas.1002143107 20410458 PMC2889350

[B18] Chanda N. Shukla R. Katti K. Kannan R. (2009). Gastrin releasing protein receptor specific gold nanorods: breast and prostate tumor avid nanovectors for molecular imaging. Nano Lett. 9, 1798–1805 10.1021/nl8037147 19351145 PMC2699898

[B19] Chapman J. Gouazé-Andersson V. Messner M. Flowers M. Karimi R. Kester M. (2010). Metabolism of short-chain ceramide by human cancer cells-Implications for therapeutic approaches. Biochem. Pharmacol. 80, 308–315 10.1016/j.bcp.2010.04.001 20385104 PMC2883648

[B21] Chavanpatil M. Khdair A. Gerard B. Bachmeier C. Miller D. Shekhar M. (2007). Surfactant-polymer nanoparticles overcome P-glycoprotein- mediated drug efflux. Mol. Pharm. 4, 730–738 10.1021/mp070024d 17705442

[B20] Chavanpatil M. Patil Y. Panyam J. (2006). Susceptibility of nanoparticle-encapsulated Paclitaxel to P-glycoprotein-mediated drug efflux. Int. J. Pharm. 320, 150–156 10.1016/j.ijpharm.2006.03.045 16713148

[B22] Chen A. Zhang M. Wei D. Stueber D. Taratula O. Minko T. (2009a). Co-delivery of Doxorubicin and Bcl-2 siRNA by mesoporous silica nanoparticles enhances the efficacy of chemotherapy in multidrug-resistant. Small 5, 2673–2677 10.1002/smll.200900621 19780069 PMC2833276

[B23] Chen B. Lai B. Cheng J. Xia G. Gao F. Xu W. (2009b). Daunorubicin-loaded magnetic nanoparticles of Fe_3_O_4_ overcome multidrug resistance and induce apoptosis of K562-n/VCR cells *in-vivo*. Int. J. Nanomedicine 4, 201–208 10.2147/IJN.S7287 19918366 PMC2775690

[B24] Chen B. Sun Q. Wang X. Gao F. Dai Y. Yin Y. (2008). Reversal in multidrug resistance by magnetic nanoparticle of Fe_3_O_4_ loaded with Adriamycin and tetrandrine in K562/A02 leukemic cells. Int. J. Nanomedicine 3, 277–286 18686787 10.2147/ijn.s2714 PMC2527663

[B25] Chen Y. Bathula S. Li J. Huang L. (2010). Multifunctional nanoparticles delivering small interfering RNA and doxorubicin overcome drug resistance in cancer. J. Biol. Chem. 285, 22639–22650 10.1074/jbc.M110.125906 20460382 PMC2903346

[B26] Chen Y. Yin Q. Ji X. Zhang S. Chen H. Zheng Y. (2012). Manganese oxide-based multifunctionalized mesoporous silica nanoparticles for pH-responsive MRI, ultrasonography and circumvention of MDR in cancer cells. Biomaterials 33, 7126–7137 10.1016/j.biomaterials.2012.06.059 22789722

[B27] Chen Y. Zhang W. Gu J. Ren Q. Fan Z. Zhong W. (2013). Enhanced antitumor efficacy by methotrexate conjugated pluronic mixed micelles against KBv multidrug resistant cancer. Int. J. Pharm. 452, 421–433 10.1016/j.ijpharm.2013.05.015 23688623

[B28] Chicheł A. Skowronek J. Kubaszewska M. Kanikowski M. (2007). Hyperthermia - description of a method and a review of clinical applications. Rep. Pract. Oncol. Radiother. 12, 267–275 10.1016/S1507-1367(10)60065-X17350947

[B29] Chiu S. Liu S. Perrotti D. Marcucci G. Lee R. (2006). Efficient delivery of a Bcl-2-specific antisense oligodeoxyribonucleotide (G3139) via transferrin receptor-targeted liposomes. J. Control. Release 112, 199–207 10.1016/j.jconrel.2006.02.011 16564596

[B30] Cho H. Yoon H. Koo H. Ko S. Shim J. Lee J. (2011). Self-assembled nanoparticles based on hyaluronic acid-ceramide (HA-CE) and Pluronic® for tumor-targeted delivery of docetaxel. Biomaterials 32, 7181–7190 10.1016/j.biomaterials.2011.06.028 21733572

[B31] Cho H. Yoon I. Yoon H. Koo H. Jin Y. Ko S. (2012). Polyethylene glycol-conjugated hyaluronic acid-ceramide self-assembled nanoparticles for targeted delivery of doxorubicin. Biomaterials 33, 1190–1200 10.1016/j.biomaterials.2011.10.064 22074664

[B32] Cho Y. Yoon T. Jang E. Hong K. Lee S. Kim O. (2010). Cetuximab-conjugated magneto-fluorescent silica nanoparticles for *in-vivo* colon cancer targeting and imaging. Cancer Lett. 299, 63–71 10.1016/j.canlet.2010.08.004 20826046

[B33] Cirstoiu-Hapca A. Buchegger F. Bossy L. Kosinski M. Gurny R. Delie F. (2009). Nanomedicines for active targeting: physicochemical characterization of Paclitaxel loaded antiHER2 immunonanoparticles and *in vitro* functional studies on target cells. Eur. J. Pharm. Sci. 38, 230–237 10.1016/j.ejps.2009.07.006 19632322

[B34] Conte C. Ungaro F. Maglio G. Tirino P. Siracusano G. Sciortino M. (2013). Biodegradable core-shell nanoassemblies for the delivery of docetaxel and Zn (II)-phthalocyanine inspired by combination therapy for cancer. J. Control. Release 167, 40–52 10.1016/j.jconrel.2012.12.026 23298613

[B35] Couvreur P. Vauthier C. (1991). Polyalkylcyanoacrylate nanoparticles as drug carrier: present state and perspectives. J. Control. Release 17, 187–198

[B36] Dantzig A. Alwis D. Burgess M. (2003). Considerations in the design and development of transport inhibitors as adjuncts to drug therapy. Adv. Drug Deliv. Rev. 55, 133–150 10.1016/S0169-409X(02)00175-8 12535578

[B37] Das M. Sahoo S. (2012). Folate decorated dual drug loaded nanoparticle: role of curcumin in enhancing therapeutic potential of nutlin-3a by reversing multidrug resistance. PLoS ONE 7:32920 10.1371/journal.pone.0032920 22470431 PMC3310050

[B38] Davis M. Zuckerman J. Choi C. (2010). Evidence of RNAi in humans from systemically administered siRNA via targeted nanoparticles. Nature 464, 1067–1070 10.1038/nature08956 20305636 PMC2855406

[B39] Devalapally H. Duan Z. Seiden M. Amiji M. (2007). Paclitaxel and ceramide co-administration in biodegradable polymeric nanoparticulate delivery system to overcome drug resistance in ovarian cancer. Int. J. Cancer. 121, 1830–1838 10.1002/ijc.22886 17557285

[B40] Devalapally H. Duan Z. Seiden M. Amiji M. (2008). Modulation of drug resistance in ovarian adenocarcinoma by enhancing intracellular ceramide using tamoxifen-loaded biodegradable polymeric nanoparticles. Clin. Cancer Res. 14, 3193 10.1158/1078-0432.CCR-07-4973 18483388

[B41] Dong X. Mumper R. (2010). Nanomedicinal strategies to treat multidrug-resistant tumors: current progress. Nanomedicine (Lond.) 5, 597–615 10.2217/nnm.10.35 20528455 PMC2925023

[B42] Emami J. Rezazadeh M. Varshosaz J. Tabbakhian M. Aslani A. (2012). Formulation of LDL targeted nanostructured lipid carriers loaded with Paclitaxel: a detailed study of preparation, freeze drying condition and *in-vitro* cytotoxicity. J. Nanomater. 2012:358782 10.1155/2012/358782

[B43] Fan L. Li F. Zhang H. Wang Y. Cheng C. Li X. (2010). Co-delivery of PDTC and doxorubicin by multifunctional micellar nanoparticles to achieve active targeted drug delivery and overcome multidrug resistance. Biomaterials 31, 5634–5642 10.1016/j.biomaterials.2010.03.066 20430433

[B44] Fang C. Kievit F. Veiseh O. Stephen Z. Wang T. Lee D. (2012). Fabrication of magnetic nanoparticles with controllable drug loading and release through a simple assembly approach. J. Control. Release 162, 233–241 10.1016/j.jconrel.2012.06.028 22735239 PMC3422574

[B45] Fardel O. Lecureur V. Guillouzo A. (1996). The P-glycoprotein multidrug transporter. Gen. Pharmacol. 27, 1283–1291 9304397 10.1016/s0306-3623(96)00081-x

[B46] Fox E. Bates S. (2007). Tariquidar (XR9576): a P-glycoprotein drug efflux pump inhibitor. Expert Rev. Anticancer Ther. 7, 447–459 10.1586/14737140.7.4.447 17428165

[B47] Fukumura D. Jain R. (2007). Tumor microvasculature and microenvironment: targets for anti-angiogenesis and normalization. Microvasc. Res. 74, 72–84 10.1016/j.mvr.2007.05.003 17560615 PMC2100036

[B48] Ganta S. Amiji M. (2009). Co-administration of Paclitaxel and Curcumin in nanoemulsion formulations to overcome multidrug resistance in tumor cells. Mol. Pharm. 6, 928–939 10.1021/mp800240j 19278222

[B49] Gao Y. Chen L. Zhang Z. Chen Y. Li Y. (2011). Reversal of multidrug resistance by reduction-sensitive linear cationic click polymer/iMDR1-pDNA complex nanoparticles. Biomaterials 32, 1738–1747 10.1016/j.biomaterials.2010.11.001 21112086

[B50] Gao Z. Fain H. Rapoport N. (2005). Controlled and targeted tumor chemotherapy by micellar-encapsulated drug and ultrasound. J. Control. Release 102, 203–222 10.1016/j.jconrel.2004.09.021 15653146

[B51] Gao Z. Zhang L. Sun Y. (2012). Nanotechnology applied to overcome tumor drug resistance. J. Control. Release 162, 45–55 10.1016/j.jconrel.2012.05.051 22698943

[B52] Gary-Bobo M. Hocine O. Brevet D. Maynadier M. Raehm L. Richeter S. (2012). Cancer therapy improvement with mesoporous silica nanoparticles combining targeting, drug delivery and PDT. Int. J. Pharm. 423, 509–515 10.1016/j.ijpharm.2011.11.045 22178618

[B53] Geszke-Moritz M. Moritz M. (2013). Quantum dots as versatile probes in medical sciences: synthesis, modification and properties. Mat. Sci. Eng. C 33, 1008–1021 10.1016/j.msec.2013.01.003 23827537

[B54] Gokhale P. Radhakrishnan B. Husain S. Abernethy D. Sacher R. Dritschilo A. (1996). An improved method of encapsulation of doxorubicin in liposomes: pharmacological, toxicological and therapeutic evaluation. Br. J. Cancer 74, 43–48 8679456 10.1038/bjc.1996.313 PMC2074597

[B55] Gong J. Chen M. Zheng Y. Wang S. Wang Y. (2012). Polymeric micelles drug delivery system in oncology. J. Control. Release 159, 312–323 10.1016/j.jconrel.2011.12.012 22285551

[B56] Gu Y. Cheng J. Man C. Wong W. Cheng S. (2012). Gold-doxorubicin nanoconjugates for overcoming multidrug resistance. Nanomedicine 8, 204–211 10.1016/j.nano.2011.06.005 21704592

[B57] Han M. Diao Y. Jiang H. Ying X. Chen D. Liang W. (2011). Molecular mechanism study of chemosensitization of doxorubicin-resistant human myelogenous leukemia cells induced by a composite polymer micelle. Int. J. Pharm. 420, 404–411 10.1016/j.ijpharm.2011.09.009 21945184

[B58] Hannun Y. Obeid L. (1995). Ceramide: an intracellular signal for apoptosis. Trends Biochem. Sci. 20, 73–77 10.1016/S0968-0004(00)88961-6 7701566

[B59] Harris A. (2002). Hypoxia - a key regulatory factor in tumor growth. Nat. Rev. Cancer 2, 38–47 10.1038/nrc704 11902584

[B60] He Q. Gao Y. Zhang L. Zhang Z. Gao F. Ji X. (2011). A pH-responsive mesoporous silica nanoparticles-based multi-drug delivery system for overcoming multi-drug resistance. Biomaterials 32, 7711–7720 10.1016/j.biomaterials.2011.06.066 21816467

[B61] Howard B. Gao Z. Lee S. (2006). Ultrasound-enhanced chemotherapy of drug-resistant breast cancer tumors by micellar-encapsulated Paclitaxel. Am. J. Drug Deliv. 4, 97–104 10.2165/00137696-200604020-00005

[B62] Hun X. Zhang Z. Tiao L. (2008). Anti-Her-2 monoclonal antibody conjugated polymer fluorescent nanoparticles probe for ovarian cancer imaging. Anal. Chim. Acta 625, 201–206 10.1016/j.aca.2008.07.038 18724995

[B63] Indran I. Tufo G. Pervaiz S. Brenner C. (2011). Recent advances in apoptosis, mitochondria and drug resistance in cancer cells. Biochim. Biophys. Acta 1807, 735–745 10.1016/j.bbabio.2011.03.010 21453675

[B64] Jagani H. Josyula V. Palanimuthu V. (2013). Improvement of therapeutic efficacy of PLGA nanoformulation of siRNA targeting anti-apoptotic Bcl-2 through chitosan coating. Eur. J. Pharm. Sci. 48, 611–618 10.1016/j.ejps.2012.12.017 23291045

[B65] Javid A. Ahmadian S. Saboury A. Rezaei-Zarchi S. (2011). Anticancer effect of doxorubicin loaded heparin based super-paramagnetic iron oxide nanoparticles against the human ovarian cancer cells. World Acad. Sci. Eng. Tech. 50

[B66] Jia L. Shen J. Li Z. Zhang D. Zhang Q. Liu G. (2013). *In-vitro* and *in-vivo* evaluation of paclitaxel-loaded mesoporous silica nanoparticles with three pore sizes. Inter. J. Pharm. 445, 12–19 23384728 10.1016/j.ijpharm.2013.01.058

[B67] Jiang Y. DiVittore N. Kaiser J. Shanmugavelandy S. Fritz J. Heakal Y. (2011). Combinatorial therapies improve the therapeutic efficacy of nanoliposomal ceramide for pancreatic cancer. Landes Biosci. 12, 574–585 10.4161/cbt.12.7.15971 21795855 PMC3218384

[B68] Jiang Z. Chen B. Wu Q. Xia G. Zhang Y. Gao F. (2009). Reversal effect of Fe_3_O_4_-magnetic nanoparticles on multi-drug resistance of ovarian carcinoma cells and its correlation with apoptosis-associated genes. Chin. J. Cancer 28, 1158–1162 10.5732/cjc.009.10090 19895735

[B69] Kang K. Chun M.-K. Kim O. Subedi R. K. Ahn S. G. Yoon J. H. (2010). Doxorubicin-loaded solid lipid nanoparticles to overcome multidrug resistance in cancer therapy. Nanomed. Nanotech. Bio. Med. 6, 210–213 10.1016/j.nano.2009.12.006 20060074

[B70] Kedar U. Phutane P. Shidhaye S. Kadam V. (2010). Advances in polymeric micelles for drug delivery and tumor targeting. Nanomedicine 6, 714–729 10.1016/j.nano.2010.05.005 20542144

[B71] Kelkar S. Reineke T. (2011). Theranostics: combining imaging and therapy. Bioconjug. Chem. 22, 1879–1903 10.1021/bc200151q 21830812

[B72] Kesharwani P. Tekade R. Gajbhiye V. Jain K. Jain M. (2011). Cancer targeting potential of some ligand-anchored poly(propylene imine) dendrimers: a comparison. Nanomed. Nanotech. Bio. Med. 7, 295–304 10.1016/j.nano.2010.10.010 21070888

[B73] Khandare J. Jayant S. Singh A. Chandna P. Wang Y. Vorsa N. (2006). Dendrimer versus linear conjugate: influence of polymeric architecture on the delivery and anticancer effect of paclitaxel. Bioconj. Chem. 17, 1464–1472 10.1021/bc060240p 17105225

[B73a] Khdair A. Handa H. Mao G. Panyam J. (2009). Nanoparticle-mediated combination chemotherapy and photodynamic therapy overcomes tumor drug resistance *in vitro*. Eur. J. Pharm. Biopharm. 71, 214–222 10.1016/j.ejpb.2008.08.017 18796331

[B74] Kievit F. Wang F. Fang C. Mok H. Wang K. Silber J. (2011). Doxorubicin loaded iron oxide nanoparticles overcome multidrug resistance in cancer *in vitro*. J. Control. Release 152, 76–83 10.1016/j.jconrel.2011.01.024 21277920 PMC3110619

[B75] Kim E. Jung Y. Choi H. Yang J. Suh J. Huh Y. (2010a). Prostate cancer cell death produced by the co-delivery of Bcl-xL shRNA and doxorubicin using an aptamer-conjugated polyplex. Biomaterials 31, 4592–4599 10.1016/j.biomaterials.2010.02.030 20206379

[B76] Kim J. Choi W. Kim M. Tae G. (2013). Tumor-targeting nanogel that can function independently for both photodynamic and photothermal therapy and its synergy from the procedure of PDT followed by PTT. J. Control. Release 171, 113–121 10.1016/j.jconrel.2013.07.006 23860187

[B77] Kim K. Kim J. Park H. Kim Y. Park K. Nam H. (2010b). Tumor-homing multifunctional nanoparticles for cancer theragnosis: simultaneous diagnosis, drug delivery, and therapeutic monitoring. J. Control. Release 146, 219–227 10.1016/j.jconrel.2010.04.004 20403397

[B78] Kirui D. Khalidov I. Wang Y. Batt C. (2013). Targeted near-IR hybrid magnetic nanoparticles for *in-vivo* cancer therapy and imaging. Nanomed. Nanotech. Bio. Med. 9, 702–711 10.1016/j.nano.2012.11.009 23219875

[B79] Kos J. Obermajer N. Doljak B. Kocbek P. Kristl J. (2009). Inactivation of harmful tumor associated proteolysis by nanoparticulate system. Int. J. Pharm. 381, 106–112 10.1016/j.ijpharm.2009.04.037 19422896

[B80] Koshkaryev A. Piroyan A. Torchilin V. (2012). Increased apoptosis in cancer cells *in vitro* and *in vivo* by ceramides in transferrin-modified liposomes. Cancer Bio. Ther. 13, 50–60 10.4161/cbt.13.1.18871 22336588 PMC3335981

[B81] Kumar A. Liang X. (2011). Gold nanoparticles: promising nanomaterials for the diagnosis of cancer and HIV/AIDS. J. Nanomater. 2011:202187 10.1155/2011/202187

[B82] Kumar A. Ma H. Zhang X. Huang K. Jin S. Liu J. (2012). Gold nanoparticles functionalized with therapeutic and targeted peptides for cancer treatment. Biomaterials 33, 1180–1189 10.1016/j.biomaterials.2011.10.058 22056754

[B83] Kumar A. Xu Z. Xing-Jie L. (2013). Gold nanoparticles: emerging paradigm for targeted drug delivery system. Biotech. Adv. 31, 593–606 10.1016/j.biotechadv.2012.10.002 23111203

[B84] Kumar C. Faruq M. (2011). Magnetic nanomaterials for hyperthermia-based therapy and controlled drug delivery. Adv. Drug Deliv. Rev. 63, 789–808 10.1016/j.addr.2011.03.008 21447363 PMC3138885

[B85] Kuo W. Ku Y. Sei H. Cheng F. Yeh C. (2009). Paclitaxel-loaded stabilizer-free poly(D,L-lactide-co-glycolide) nanoparticles conjugated with quantum dots for reversion of anti-cancer drug resistance and cancer cellular imaging. J. Chin. Chem. Soc. 56, 923–934 10.1002/jccs.200900136

[B85a] Lage H. (2009). Therapeutic potential of RNA interference in drug-resistant cancers. Future Oncol. 5, 169–185 10.2217/14796694.5.2.169 19284376

[B86] Lee D. Lockey R. Mohapatra S. (2006). Folate receptor-mediated cancer cell specific gene delivery using folic acid-conjugated oligochitosans. J. Nanosci. Nanotechnol. 6, 2860–2866 10.1166/jnn.2006.465 17048492

[B87] Lee E. Na K. Bae Y. (2005). Doxorubicin loaded pH-sensitive polymeric micelles for reversal of resistant MCF-7 tumor. J. Control. Release 103, 405–418 10.1016/j.jconrel.2004.12.018 15763623

[B88] Lee H. Kim S. Choi B. Park M. Lee J. Jeong S. (2011). Hyperthermia improves therapeutic efficacy of doxorubicin carried by mesoporous silica nanocontainers in human lung cancer cells. Int. J. Hyperthermia 27, 698–707 10.3109/02656736.2011.608217 21992562

[B89] Lee R. Low P. (1995). Folate-mediated tumor cell targeting of liposome-entrapped doxorubicin *in-vitro*. Biochim. Biophys. Acta Biomembranes 1233, 134–144 10.1016/0005-2736(94)00235-H 7865538

[B90] Li B. Xu H. Li Z. Yao M. Xie M. Shen H. (2012a). Bypassing multidrug resistance in human breast cancer cells with lipid/polymer particle assemblies. Int. J. Nanomedicine 7, 187–197 10.2147/IJN.S27864 22275834 PMC3263411

[B91] Li F. Sethi G. (2010). Targeting transcription factor NF-κB to overcome chemoresistance and radioresistance in cancer therapy. Biochim. Biophys. Acta 1805, 167–180 10.1016/j.bbcan.2010.01.002 20079806

[B92] Li K. Chen B. Xu L. Feng J. Xia G. Cheng J. (2013a). Reversal of multidrug resistance by cisplatin-loaded magnetic Fe3O4 nanoparticles in A549/DDP lung cancer cells *in-vitro* and *in-vivo*. Int. J. Nanomedicine 8, 1867–1877 10.2147/IJN.S43752 23690684 PMC3656817

[B93] Li M.-H. Choi S. Thomas T. Desai A. Lee K.-H. Holl M. (2012b). Dendrimer-based multivalent methotrexates as dual acting nanoconjugates for cancer cell targeting. Eur. J. Med. Chem. 47, 560–572 10.1016/j.ejmech.2011.11.027 22142685 PMC3243070

[B94] Li P. Zhou G. Zhu X. Li G. Yan P. Shen L. (2012c). Photodynamic therapy with hyperbranched poly(ether-ester) chlorin(e6) nanoparticles on human tongue carcinoma CAL-27 cells. Photodiagnosis Photodyn. Ther. 9, 76–82 10.1016/j.pdpdt.2011.08.001 22369732 PMC3292741

[B95] Li X. Chen Y. Wang M. Ma Y. Xia W. Gu H. (2013b). A mesoporous silica nanoparticle – PEI – fusogenic peptide system for siRNA delivery in cancer therapy. Biomaterials 34, 1391–1401 10.1016/j.biomaterials.2012.10.072 23164421

[B96] Li X. Xie Q. Zhang J. Xia W. Gua H. (2011). The packaging of siRNA within the mesoporous structure of silica nanoparticles. Biomaterials 32, 9546–9556 10.1016/j.biomaterials.2011.08.068 21906804

[B97] Ling Y. Wei K. Zou F. Zhong S. (2012). Temozolomide loaded PLGA-based superparamagnetic nanoparticles for magnetic resonance imaging and treatment of malignant glioma. Int. J. Pharm. 430, 266–275 10.1016/j.ijpharm.2012.03.047 22486964

[B98] Liu Q. Zhang J. Xia W. Gu H. (2012). Magnetic field enhanced cell uptake efficiency of magnetic silica mesoporous nanoparticles. Nanoscale 4, 3415–3421 10.1039/c2nr30352c 22543531

[B99] Liu X. Ryland L. Yang J. Liao A. Aliaga C. Watts R. (2010). Targeting of survivin by nanoliposomal ceramide induces complete remission in a rat model of NK-LGL leukemia. Blood 116, 4192–4201 10.1182/blood-2010-02-271080 20671121 PMC2993625

[B100] Liu X. Sun J. (2010). Endothelial cells dysfunction induced by silica nanoparticles through oxidative stress via JNK/P53 and NF-κB pathways. Biomaterials 31, 8198–8209 10.1016/j.biomaterials.2010.07.069 20727582

[B101] Liu Y. Lillehei K. Cobb W. Christians U. Ng K. (2001). Overcoming MDR by ultrasound-induced hyperthermia and P-glycoprotein modulation. Biochem. Biophys. Res. Commun. 289, 62–68 10.1006/bbrc.2001.5938 11708777

[B102] Lu F. Doane T. Zhu J. Burda C. (2012). Gold nanoparticles for diagnostic sensing and therapy. Inorg. Chim. Acta 393, 142–153 10.1016/j.ica.2012.05.038

[B103] Lu H. Syu W. Nishiyama N. Kataoka K. Lai P. (2011). Dendrimer phthalocyanine-encapsulated polymeric micelle-mediated photochemical internalization extends the efficacy of photodynamic therapy and overcomes drug-resistance *in-vivo*. J. Control. Release 155, 458–464 10.1016/j.jconrel.2011.06.005 21689700

[B104] Luk B. Fang R. Zhang L. (2012). Lipid and polymer-based nanostructures for cancer theranostics. Theranostics 2, 1117–1126 10.7150/thno.4381 23382770 PMC3563151

[B105] Luo C. Zhu Y. Jiang T. Lu X. Zhang W. Jing Q. (2007). Matrine induced gastric cancer MKN45 cells apoptosis via increasing pro-apoptotic molecules of Bcl-2 family. Toxicology 229, 245–252 10.1016/j.tox.2006.10.020 17134813

[B106] Luqman S. Pezzuto J. (2010). NF-κB: a promising target for natural products in cancer chemoprevention. Phytother. Res. 24, 949–963 10.1002/ptr.3171 20577970

[B107] Ma P. Benhabbour S. Feng L. Mumper R. (2013). 2′-Behenoyl-paclitaxel conjugate containing lipid nanoparticles for the treatment of metastatic breast cancer. Cancer Lett. 334, 253–262 10.1016/j.canlet.2012.08.009 22902506 PMC3522796

[B108] Ma P. Dong X. Swadley C. Gupte A. Leggas M. Ledeburand H. (2009). Development of idarubicin and doxorubicin solid lipid nanoparticles to overcome P-gp mediated multiple drug resistance in leukemia. J. Biomed. Nanotechnol. 5, 151–161 10.1166/jbn.2009.1021 20055093 PMC2805476

[B109] MacDiarmid J. Amaro-Mugridge N. Madrid-Weiss J. Sedliarou I. Wetzel S. Kochar K. (2009). Sequential treatment of drug-resistant tumors with targeted minicells containing siRNA or a cytotoxic drug. Nat. Biotechnol. 27, 643–651 10.1038/nbt.1547 19561595

[B110] Maeng J. Lee D. Jung K. Bae Y. Park I. Jeong S. (2010). Multifunctional doxorubicin loaded superparamagnetic iron oxide nanoparticles for chemotherapy and magnetic resonance imaging in liver cancer. Biomaterials 31, 4995–5006 10.1016/j.biomaterials.2010.02.068 20347138

[B111] Magné N. Toillon R. Bottero V. Didelot C. Van Houtte P. Gérard J. (2006). NF-κB modulation and ionizing radiation: mechanisms and future directions for cancer treatment. Cancer Lett. 231, 158–168 10.1016/j.canlet.2005.01.022 16399220

[B113] Mai W. Meng H. (2013). Mesoporous silica nanoparticles: a multifunctional nanotherapeutic system. Integr. Biol. (Camb.) 5, 19–28 10.1039/c2ib20137b 23042147

[B114] Majd M. Asgari D. Barar J. Valizadeh H. Kafil V. Abadpour A. (2013). Tamoxifen loaded folic acid armed PEGylated magnetic nanoparticles for targeted imaging and therapy of cancer. Colloids Surf. B Biointerfaces 106, 117–125 10.1016/j.colsurfb.2013.01.051 23434700

[B115] Mamaeva V. Sahlgren C. Lindén M. (2013). Mesoporous silica nanoparticles in medicine-recent advances. Adv. Drug Deliv. Rev. 65, 689–702 10.1016/j.addr.2012.07.018 22921598

[B116] Marin A. Sun H. Husseini G. Pitt W. Christensen D. Rapoport N. (2002). Drug delivery in pluronic micelles: effect of high-frequency ultrasound on drug release from micelles and intracellular uptake. J. Control. Release 84, 39–47 10.1016/S0168-3659(02)00262-6 12399166

[B117] Mei L. Zhang Y. Zheng Y. Tian G. Song C. Yang D. (2009). A novel docetaxel-loaded poly (ε-Caprolactone)/Pluronic F68 nanoparticle overcoming multidrug resistance for breast cancer treatment. Nanoscale Res. Lett. 4, 1530–1539 10.1007/s11671-009-9431-6 20652101 PMC2894322

[B118] Mendoza A. Préat V. Mollinedo F. Blanco-Prieto M. (2011). *In-vitro* and *in-vivo* efficacy of edelfosine-loaded lipid nanoparticles against glioma. J. Control. Release 156, 421–426 10.1016/j.jconrel.2011.07.030 21821074

[B119] Miao J. Du Y. Yuan H. Zhang X. Hu F. (2013). Drug resistance reversal activity of anticancer drug loaded solid lipid nanoparticles in multi-drug resistant cancer cells. Colloids Surf. B Biointerfaces 110, 74–80 10.1016/j.colsurfb.2013.03.037 23711779

[B120] Miglietta A. Cavalli R. Bocca C. Gabriel L. Gasco M. (2000). Cellular uptake and cytotoxicity of solid lipid nanospheres (SLN) incorporating doxorubicin or paclitaxel. Int. J. Pharm. 210, 61–67 10.1016/S0378-5173(00)00562-7 11163988

[B121] Milane L. Ganesh S. Shah S. Duan Z. Amiji M. (2011). Multi-modal strategies for overcoming tumor drug resistance: hypoxia, the Warburg effect, stem cells and multifunctional nanotechnology. J. Control. Release 155, 237–247 10.1016/j.jconrel.2011.03.032 21497176 PMC3146561

[B122] Min K. Park K. Kim Y. Bae S. Lee S. Jo H. (2008). Hydrophobically modified glycol chitosan nanoparticles-encapsulated camptothecin enhance the drug stability and tumor targeting in cancer therapy. J. Control. Release 127, 208–218 10.1016/j.jconrel.2008.01.013 18336946

[B123] Morad S. Madigan J. Levin J. Abdelmageed N. Karimi R. Rosenberg D. (2013). Tamoxifen magnifies therapeutic impact of ceramide in human colorectal cancer cells independent of p53. Biochem. Pharmacol. 85, 1057–1065 10.1016/j.bcp.2013.01.015 23353700 PMC3604153

[B124] Na J. Koo H. Lee S. Min K. Park K. Yoo H. (2011). Real-time and non-invasive optical imaging of tumor-targeting glycol chitosan nanoparticles in various tumor models. Biomaterials 32, 5252–5261 10.1016/j.biomaterials.2011.03.076 21513975

[B125] Nadali F. Pourfathollah A. Alimoghaddam C. (2007). Multidrug resistance inhibition by antisense oligonucleotide against MDR1/mRNA in P-glycoprotein expressing leukemic cells. Hematology 12, 393–401 10.1080/10245330701283991 17852455

[B126] Nair H. Anand P. Sung B. Kunnumakkara A. Yadav V. Tekmal R. (2010). Design of curcumin-loaded PLGA nanoparticles formulation with enhanced cellular uptake, and increased bioactivity *in vitro* and superior bioavailability *in vivo*. Biochem. Pharmacol. 79, 330–338 10.1016/j.bcp.2009.09.003 19735646 PMC3181156

[B127] Némati F. Dubernet C. Fessi H. Colin de Verdière A. Poupon M. Puisieux F. (1996). Reversion of multidrug resistance using nanoparticles in vitro: influence of the nature of the polymer. Int. J. Pharm. 138, 237–246 10.1016/0378-5173(96)04559-0

[B128] Newell B. Wang Y. Irudayaraj J. (2012). Multifunctional gold nanorod theragnostics probed by multi-photon imaging, Eur. J. Med. Chem. 48, 330–337 10.1016/j.ejmech.2011.12.036 22230223

[B128a] Nieth C. Priebsch A. Stege A. Lage H. (2003). Modulation of the classical multidrug resistance (MDR) phenotype by RNA interference (RNAi). FEBS Lett. 545, 144–150 10.1016/S0014-5793(03)00523-4 12804765

[B129] Nishiyama N. Nakagishi Y. Morimoto Y. Lai P. Miyazaki K. Urano K. (2009). Enhanced photodynamic cancer treatment by supramolecular nanocarriers charged with dendrimer phthalocyanine. J. Control. Release 133, 245–251 10.1016/j.jconrel.2008.10.010 19000725

[B130] Noguchi S. Hirashima N. Furuno T. Nakanishi M. (2003). Remarkable induction of apoptosis in cancer cells by a novel cationic liposome complexed with a bcl-2 antisense oligonucleotide. J. Control. Release 88, 313–320 10.1016/S0168-3659(02)00484-4 12628337

[B131] Nombona N. Maduray K. Antunes E. Karsten A. Nyokong T. (2012). Synthesis of phthalocyanine conjugates with gold nanoparticles and liposomes for photodynamic therapy. J. Photochem. Photobiol. B Biol. 107, 35–44 10.1016/j.jphotobiol.2011.11.007 22209036

[B132] O'Donnell J. Joyce M. Shannon A. Harmey J. Geraghty J. Bouchier-Hayes D. (2006). Oncological implications of hypoxia inducible factor-1α (HIF-1α) expression. Cancer Treat. Rev. 32, 407–416 10.1016/j.ctrv.2006.05.003 16889900

[B133] Okayama R. Noji M. Nakanishi M. (1997). Cationic cholesterol with a hydroxyethylamino head group promotes significantly liposome-mediated gene transfection. FEBS Lett. 408, 232–234 10.1016/S0014-5793(97)00431-6 9187373

[B134] Otranto M. Sarrazy V. Bonte F. Hinz B. Gabbiani G. Desmouliere A. (2012). The role of the myofibroblast in tumor stroma remodelling. Cell Adh. Migr. 6, 1–49 10.4161/cam.20377 22568985 PMC3427235

[B135] Ozben T. (2006). Mechanisms and strategies to overcome multiple drug resistance in cancer. FEBS Lett. 580, 2903–2909 10.1016/j.febslet.2006.02.020 16497299

[B136] Pan L. Liu J. He Q. Wang L. Shi J. (2013). Overcoming multidrug resistance of cancer cells by direct intranuclear drug delivery using TAT-conjugated mesoporous silica nanoparticles. Biomaterials 34, 2719–2730 10.1016/j.biomaterials.2012.12.040 23337327

[B137] Paszko E. Vaz G. Ehrhardt C. Senge M. (2013). Transferrin conjugation does not increase the efficiency of liposomal Foscan during *in vitro* photodynamic therapy of oesophageal cancer. Eur. J. Pharm. Sci. 48, 202–210 10.1016/j.ejps.2012.10.018 23159666

[B138] Patil Y. Sadhukh T. Ma L. Panyam T. (2009b). Nanoparticle-mediated simultaneous and targeted delivery of paclitaxel and tariquidar overcomes tumor drug resistance. J. Control. Release 136, 21–29 10.1016/j.jconrel.2009.01.021 19331851

[B139] Patil Y. Swaminathan S. Sadhukha T. Ma L. Panyam J. (2010). The use of nanoparticle-mediated targeted gene silencing and drug delivery to overcome tumor drug resistance. Biomaterials 31, 358–365 10.1016/j.biomaterials.2009.09.048 19800114 PMC2783414

[B140] Patil Y. Toti U. Khdair A. Ma L. Panyam J. (2009a). Single step surface functionalization of polymeric nanoparticles for targeted drug delivery. Biomaterials 30, 859–866 10.1016/j.biomaterials.2008.09.056 19019427 PMC2637351

[B141] Pettus B. Chalfant C. Hannun Y. (2002). Ceramide in apoptosis: an overview and current perspectives. Biochim. Biophys. Acta 1585, 114–125 10.1016/S1388-1981(02)00331-1 12531544

[B142] Pramanik D. Campbell N. Das S. Gupta S. Chenna V. Bisht S. (2012). A composite polymer nanoparticle overcomes multidrug resistance and ameliorates doxorubicin-associated cardiomyopathy. Oncotarget 3, 640–650 22791660 10.18632/oncotarget.543 PMC3442295

[B143] Prasad P. Shuhendler A. Cai P. Rauth A. Wu X. (2013). Doxorubicin and mitomycin C co-loaded polymer-lipid hybrid nanoparticles inhibit growth of sensitive and multidrug resistant human mammary tumor xenografts. Cancer Lett. 334, 263–273 10.1016/j.canlet.2012.08.008 22902994

[B144] Rapisarda A. Melillo G. (2009). Role of the hypoxic tumor microenvironment in the resistance to anti-angiogenic therapies. Drug Resist. Update 12, 74–80 10.1016/j.drup.2009.03.002 19394890 PMC2696589

[B145] Rapoport N. (2004). Combined cancer therapy by micellar-encapsulated drug and ultrasound. Int. J. Pharm. 277, 155–162 10.1016/j.ijpharm.2003.09.048 15158978

[B146] Ren Y. Wang Y. Zhang Y. Wei D. (2008). Overcoming multidrug resistance in human carcinoma cells by an antisense-oligodeoxynucleotide-doxorubicin conjugate *in vitro* and *in vivo*. Mol. Pharm. 5, 579–587 10.1021/mp800001j 18461970

[B147] Robey R. Shukla S. Finley E. Oldham R. Barnett D. Ambudkar S. (2008). Inhibition of P-glycoprotein (ABCB1) - and multidrug resistance-associated protein 1 (ABCC1)-mediated transport by the orally administered inhibitor, CBT-1®. Biochem. Pharmacol. 75, 1302–1312 10.1016/j.bcp.2007.12.001 18234154 PMC2346578

[B148] Rofstad E. (2000). Microenvironment-induced cancer metastasis. Int. J. Radiat. Biol. 76, 589–605 10.1080/095530000138259 10866281

[B149] Ronaldson P. Ashraf T. Bendayan R. (2010). Regulation of multidrug resistance protein 1 by tumor necrosis factor alpha in cultured glial cells: involvement of nuclear factor-kappaB and c-Jun N-terminal kinase signaling pathways. Mol. Pharmacol. 77, 644–659 10.1124/mol.109.059410 20051532

[B150] Ryu J. Kim S. Koo H. Yhee J. Lee A. Na J. (2011). Cathepsin B-sensitive nanoprobe for *in vivo* tumor diagnosis. J. Mater. Chem. 21, 17631–17634 10.1039/c1jm13064a

[B149a] Saad M. Garbuzenko O. Minko T. (2008). Co-delivery of siRNA and an anticancer drug for treatment of multidrug-resistant cancer. Nanomedicine (Lond) 3, 761–776 10.2217/17435889.3.6.761 19025451 PMC2628713

[B150d] Sadeghi-Aliabadi H. Mozaffari M. Behdadfar B. Raesizadeh M. Zarkesh-Esfahani H. (2013). Preparation and cytotoxic evaluation of magnetite (Fe_3_O_4_) nanoparticles on breast cancer cells and its combinatory effects with doxorubicin used in hyperthermia. Avicenna J. Med. Biotechnol. 5, 96–103 23799178 PMC3689562

[B150b] Sahay G. Kim J. Kabanov A. (2010). The exploitation of differential endocytic pathways in normal and tumor cells in the selective targeting of nanoparticulate chemotherapeutic agents. Biomat 31, 923–933 10.1016/j.biomaterials.2009.09.101 19853293 PMC3082844

[B150g] Santhosh P. Ulrih N. (2013). Multifunctional superparamagnetic iron oxide nanoparticles: promising tools in cancer theranostics. Cancer Lett. 336, 8–17 10.1016/j.canlet.2013.04.032 23664890

[B152a] Savla R. Taratula O. Garbuzenko O. Minko T. (2011). Tumor targeted quantum dot-mucin 1 aptamer-doxorubicin conjugate for imaging and treatment of cancer. J. Control Rel. 153, 16–22 10.1016/j.jconrel.2011.02.015 21342659

[B152b] Schleich N. Sibret P. Danhier P. Ucakar B. Laurent S. Muller R. (2013). Dual anticancer drug/superparamagnetic iron oxide loaded PLGA-based nanoparticles for cancer therapy and magnetic resonance imaging. Int. J. Pharm. 447, 94–101 10.1016/j.ijpharm.2013.02.042 23485340

[B150a] Schornack P. Gillies R. (2003). Contributions of cell metabolism and H^+^ diffusion to the acidic pH of tumors. Neoplasia 5, 135–145 10.1016/S1476-5586(03)80005-2 12659686 PMC1502399

[B150f] Schreck R. Meier B. Männel D. Dröge W. Baeuerle P. (1992). Dithiocarbamates as potent inhibitors of nuclear factor kappa B activation in intact cells. J. Exp. Med. 175, 1181–1194 10.1084/jem.175.5.1181 1314883 PMC2119220

[B150e] Schwartz S. Hernandez A. Evers B. (1999). The role of NF-κB/IκB proteins in cancer: implications for novel treatment strategies. Surg. Oncol. 8, 143–153 10.1016/S0960-7404(00)00012-8 11113665

[B150c] Sekhon B. (2012). Current scenario on impact of nanomedicine. J. Pharm. Educ. Res. 3, 71–76

[B151a] Shabbits J. Mayer L. (2003). Intracellular delivery of ceramide lipids via liposomes enhances apoptosis *in vitro*. Biochim. Biophys. Acta. 1612, 98–106 10.1016/S0005-2736(03)00108-1 12729935

[B151c] Sharma A. K. Zhang L. Li S. Kelly D. L. Alakhov V. Y. Batrakova E. V. (2008). Prevention of MDR development in leukemia cells by micelle-forming polymeric surfactant. J. Control Rel. 131, 220–227 10.1016/j.jconrel.2008.07.031 18722489 PMC2711209

[B151] Sharom F. (1997). The P-glycoprotein efflux pump: how does it transport drugs? J. Membr. Biol. 160, 161–175 10.1007/s002329900305 9425600

[B152] Shen J. He Q. Gao Y. Shi J. Li Y. (2011). Mesoporous silica nanoparticles loading doxorubicin reverse multidrug resistance: performance and mechanism. Nanoscale 3, 4314–4322 10.1039/c1nr10580a 21892492

[B153] Shen J. Yin Q. Chen L. Zhang Z. Li Y. (2012). Co-delivery of paclitaxel and survivin shRNA by pluronic P85-PEI/TPGS complex nanoparticles to overcome drug resistance in lung cancer. Biomaterials 33, 8613–8624 10.1016/j.biomaterials.2012.08.007 22910221

[B154] Siddiqui A. Patwardhan G. Liu Y. Nazzal S. (2010). Mixed backbone antisense glucosylceramide synthase oligonucleotide (MBO-asGCS) loaded solid lipid nanoparticles: *in vitro* characterization and reversal of multidrug resistance in NCI/ADR-RES cells. Int. J. Pharm. 400, 251–259 10.1016/j.ijpharm.2010.08.044 20816930 PMC2952652

[B155] Siegal T. Horowitz A. Gabizon A. (1995). Doxorubicin encapsulated in sterically stabilized liposomes for the treatment of a brain tumor model: biodistribution and therapeutic efficacy. J. Neurosurg. 83, 1029–1037 10.3171/jns.1995.83.6.1029 7490617

[B156] Simon S. Roy D. Schindler M. (1994). Intracellular pH and the control of multi-drug resistance. Proc. Natl. Acad. Sci. U.S.A. 91, 1128–1132 10.1073/pnas.91.3.1128 8302842 PMC521467

[B157] Singh A. Fahima D. Sahoo S. (2011). Long circulating lectin conjugated paclitaxel loaded magnetic nanoparticles: a new theranostic avenue for leukemia therapy. PLoS ONE 6:e26803 10.1371/journal.pone.0026803 22110595 PMC3217954

[B158] Singh S. Kaur H. (in press). Tumor microenvironment: a review. J. Oral Maxillofac. Surg. Med. Pathol. 10.1016/j.ajoms.2012.12.011

[B159] Song X. Cai Z. Zheng Y. He G. Cui F. Gong D. (2009). Reversion of multidrug resistance by co-encapsulation of vincristine and verapamil in PLGA nanoparticles. Eur. J. Pharm. Sci. 37, 300–305 10.1016/j.ejps.2009.02.018 19491019

[B160] Stover T. Kim Y. Lowe T. Kester M. (2008). Thermoresponsive and biodegradable linear-dendritic nanoparticles for targeted and sustained release of a pro-apoptotic drug. Biomaterials 29, 359–369 10.1016/j.biomaterials.2007.09.037 17964645

[B161] Susa M. Iyer A. Ryu K. Choy E. Hornicek F. Mankin H. (2010). Inhibition of ABCB1 (MDR1) expression by a siRNA nanoparticulate delivery system to overcome drug resistance in osteosarcoma. PLoS ONE 5:10764 10.1371/journal.pone.0010764 20520719 PMC2875382

[B162] Tang M. Lei L. Guo S. Huang W. (2010). Recent progress in nanotechnology for cancer therapy. Chin. J. Cancer 29, 775–780 10.5732/cjc.010.10075 20800018

[B163] Taratula O. Kuzmov A. Shah M. Garbuzenko O. Minko T. (2013). Nanostructured lipid carriers as multifunctional nanomedicine platform for pulmonary co-delivery of anticancer drugs and siRNA. J. Control. Release 171, 349–357 10.1016/j.jconrel.2013.04.018 23648833 PMC3766401

[B164] Tas S. Vervoordeldonk M. Tak P. (2009). Gene therapy targeting nuclear factor-κB: towards clinical application in inflammatory diseases and cancer. Curr. Gene Ther. 9,160–170 10.2174/156652309788488569 19519361 PMC2864453

[B165] Teng I. Chang Y. Wang L. Lu H. Wu L. Yang C. (2013). Phospholipid-functionalized mesoporous silica nanocarriers for selective photodynamic therapy of cancer. Biomaterials 34, 7462–7470 10.1016/j.biomaterials.2013.06.001 23810081

[B166] Teow H. Zhou Z. Najlah M. Yusof S. Abbott N. D'Emanuele A. (2013). Delivery of Paclitaxel across cellular barriers using a dendrimer-based nanocarrier. Int. J. Pharm. 441, 701–711 10.1016/j.ijpharm.2012.10.024 23089576

[B167] Thakor A. Jokerst J. Zavaleta C. Massoud T. Gambhir S. (2011). Gold nanoparticles: a revival in precious metal administration to patients. Nano Lett. 11, 4029–4036 10.1021/nl202559p 21846107 PMC3195547

[B168] Thomas C. Ferris D. Lee J. Choi E. Cho M. Kim E. (2010). Noninvasive remote-controlled release of drug molecules *in vitro* using magnetic actuation of mechanized nanoparticles. J. Am. Chem. Soc. 132, 10623–10625 10.1021/ja1022267 20681678

[B169] Thomas H. Coley H. (2003). Overcoming multidrug resistance in cancer: an update on the clinical strategy of inhibiting p-glycoprotein. Cancer Control 10, 159–165 12712010 10.1177/107327480301000207

[B170] Tomida A. Tsuruo T. (2002). Drug resistance pathways as targets, in Anticancer Drug Development, Chapter 5, eds Baguley B. C. Kerr D. J. (Tokyo: Academic Press), 77–90

[B171] van Kempen L. Ruiter D. van Muijen G. Coussens L. (2003). The tumor microenvironment: a critical determinant of neoplastic evolution. Eur. J. Cell Biol. 82, 539–548 10.1078/0171-9335-00346 14703010

[B172] van Vlerken L. Duan Z. Little S. Seiden M. Amiji M. (2008). Biodistribution and pharmacokinetic analysis of paclitaxel and ceramide administered in multifunctional polymer-blend nanoparticles in drug resistant breast cancer model. Mol. Pharm. 5, 516–526 10.1021/mp800030k 18616278 PMC2646668

[B173] van Vlerken L. Duan Z. Little S. Seiden M. Amiji M. (2010). Augmentation of therapeutic efficacy in drug-resistant tumor models using ceramide coadministration in temporal-controlled polymer-blend nanoparticle delivery systems. AAPS J. 12, 171–180 10.1208/s12248-010-9174-4 20143195 PMC2844507

[B174] Vekariya K. Kaur J. Tikoo K. (2012). ERα signaling imparts chemotherapeutic selectivity to selenium nanoparticles in breast cancer. Nanomedicine 8, 1125–1132 10.1016/j.nano.2011.12.003 22197727

[B175] Velingkar V. Dandekar V. (2010). Modulation of P-glycoprotein mediated multidrug resistance (MDR) in cancer using chemosensitizers. Int. J. Pharm. Sci. Res. 1, 104–111

[B176] Venishetty V. Komuravelli R. Kuncha M. Sistla R. Diwan P. (2013). Increased brain uptake of docetaxel and ketoconazole loaded folate-grafted solid lipid nanoparticles. Nanomed. Nanotechnol. Biol. Med. 9, 111–121 10.1016/j.nano.2012.03.003 22426195

[B177] Vigderman L. Zubarev E. (2012). Therapeutic platforms based on gold nanoparticles and their covalent conjugates with drug molecules. Adv. Drug Deliv. Rev. 65, 663–676 10.1016/j.addr.2012.05.004 22613038

[B178] Wan C. Jackson J. Pirmoradi F. Chiao M. Burt H. (2012). Increased accumulation and retention of micellar Paclitaxel in drug-sensitive and P-glycoprotein-expressing cell lines following ultrasound exposure. Ultrasound Med. Bio. 38, 736–744 10.1016/j.ultrasmedbio.2012.01.023 22425383

[B179] Wang F. Zhang D. Zhang Q. Chen Y. Zheng D. Hao L. (2011a). Synergistic effect of folate-mediated targeting and verapamil-mediated P-gp inhibition with paclitaxel-polymer micelles to overcome multi-drug resistance. Biomaterials 32, 9444–9456 10.1016/j.biomaterials.2011.08.041 21903258

[B180] Wang H. Zheng L. Peng C. Guo R. Shen M. Shi X. (2011b). Computed tomography imaging of cancer cells using acetylated dendrimer-entrapped gold nanoparticles. Biomaterials 32, 2979–2988 10.1016/j.biomaterials.2011.01.001 21277019

[B181] Wang J. Tao X. Zhang Y. Wei D. Ren Y. (2010). Reversion of multidrug resistance by tumor targeted delivery of antisense oligodeoxynucleotides in hydroxypropyl-chitosan nanoparticles. Biomaterials 31, 4426–4433 10.1016/j.biomaterials.2010.02.007 20188412

[B182] Wang L. Zeng R. Li C. Qiao R. (2009). Self-assembled polypeptide block poly (vinylpyrrolidone) as prospective drug delivery systems. Colloid Surf. B Biointerfaces 74, 284–292 10.1016/j.colsurfb.2009.07.032 19717289

[B183] Wang X. Zhang R. Wu C. Dai Y. Song M. Gutmann S. (2007). The application of Fe3O4 nanoparticles in cancer research: a new strategy to inhibit drug resistance. J. Biomed. Mater. Res. A 80, 852–860 10.1002/jbm.a.30901 17072850

[B184] Weber C. Kuo P. (2012). The tumor microenvironment. Surg. Oncol. 21, 172–177 10.1016/j.suronc.2011.09.001 21963199

[B185] Wei Z. Hao J. Yuan S. Li Y. Juan W. Shaa X. (2009). Paclitaxel-loaded Pluronic P123/F127 mixed polymeric micelles: formulation, optimization and *in vitro* characterization. Int. J. Pharm. 376, 176–185 10.1016/j.ijpharm.2009.04.030 19409463

[B186] Wei Z. Yuan S. Chen Y. Yu S. Hao J. Luo J. (2010). Enhanced antitumor efficacy by Paclitaxel-loaded Pluronic P123/F127 mixed micelles against non-small cell lung cancer based on passive tumor targeting and modulation of drug resistance. Eur. J. Pharm. Biopharm. 75, 341–353 10.1016/j.ejpb.2010.04.017 20451605

[B187] Wong H. Bendayan R. Rauth A. Wu X. (2006). Simultaneous delivery of doxorubicin and GG918 (Elacridar) by new polymer-lipid hybrid nanoparticles (PLN) for enhanced treatment of multidrug-resistant breast cancer. J. Control. Release 116, 275–284 10.1016/j.jconrel.2006.09.007 17097178

[B188] Wouters B. Koritzinsky M. Chiu R. Theys J. (2003). Modulation of cell death in the tumor microenvironment. Semin. Radiat. Oncol. 13, 31–41 10.1053/srao.2003.50004 12520462

[B189] Wu C. Calcagno A. Ambudkar S. (2008). Reversal of ABC drug transporter-mediated multidrug resistance in cancer cells: evaluation of current strategies. Curr. Mol. Pharmacol. 1, 93–105 10.2174/1874467210801020093 19079736 PMC2600768

[B190] Wu F. Shao Z. Zhai B. Zhao C. Shen D. (2011). Ultrasound reverses multidrug resistance in human cancer cells by altering gene expression of ABC transporter proteins and Bax protein. Ultrasound Med. Biol. 37, 151–159 10.1016/j.ultrasmedbio.2010.10.009 21084157

[B191] Wu J. Lu Y. Lee A. Pan X. Yang X. Zhao X. (2007). Reversal of multidrug resistance by transferrin-conjugated liposomes co-encapsulating doxorubicin and verapamil. J. Pharmacol. Pharm. Sci. 10, 350–357 17727798

[B192] Xiao J. Duan X. Yin Q. Miao Z. Yu H. Chen C. (2013). The inhibition of metastasis and growth of breast cancer by blocking the NF-κB signaling pathway using bioreducible PEI-based/p65 shRNA complex nanoparticles. Biomaterials 34, 5381–5390 10.1016/j.biomaterials.2013.03.084 23591394

[B193] Xie J. Lee S. Chen X. (2010). Nanoparticle-based theranostic agents. Adv. Drug Deliv. Rev. 62, 1064–1079 10.1016/j.addr.2010.07.009 20691229 PMC2988080

[B194] Xu Z. Chen L. Gu W. Gao Y. Lin L. Zhang Z. (2009). The performance of docetaxel-loaded solid lipid nanoparticles targeted to hepatocellular carcinoma. Biomaterials 30, 226–232 10.1016/j.biomaterials.2008.09.014 18851881

[B195] Yabbarov N. Posypanova G. Vorontsov E. Obydenny S. Severin E. (2013). A new system for targeted delivery of doxorubicin into tumor cells. J. Control. Release 168, 135–141 10.1016/j.jconrel.2013.03.007 23517785

[B196] Yanan W. Baoan C. Jian C. Feng G. Wenlin X. Jiahua D. (2009). Reversal of multidrug resistance by magnetic Fe_3_O_4_ nanoparticle copolymerisating daunorubicin and 5-bromotetrandrine in xenograft nude-mice. Int. J. Nanomedicine 4, 73–78 19421372 10.2147/ijn.s5093 PMC2720736

[B197] Yang W. Cheng Y. Xu T. Wang X. Wen L. (2009a). Targeting cancer cells with biotin–dendrimer conjugates. Eur. J. Med. Chem. 44, 862–868 10.1016/j.ejmech.2008.04.021 18550227

[B198] Yang X. Koh C. Liu S. Pan X. Santhanam R. Yu B. (2009b). Transferrin receptor-targeted lipid nanoparticles for delivery of an antisense oligodeoxyribonucleotide against Bcl-2. Mol. Pharm. 6, 221–230 10.1021/mp800149s 19183107 PMC3608852

[B199] Yared J. Tkaczuk K. (2012). Update on taxane development: new analogs and new formulations. Drug Des. Devel. Ther. 6, 371–384 23251087 10.2147/DDDT.S28997 PMC3523563

[B200] Yoo M. Park I. Lim H. Lee S. Jiang H. Kim Y. (2012). Folate–PEG–superparamagnetic iron oxide nanoparticles for lung cancer imaging. Acta Biomater. 8, 3005–3013 10.1016/j.actbio.2012.04.029 22543005

[B201] Yu D. Wu F. Liu Y. Liu H. Xia Q. (2013). Bcl-2 gene silence enhances the sensitivity toward 5-Fluorouracil in gastric adenocarcinoma cells. Biomed. Pharmacother. 67, 615–617 10.1016/j.biopha.2013.03.007 23684481

[B202] Zhang G. Shi L. Selke M. Wang X. (2011a). CdTe quantum dots with daunorubicin induce apoptosis of multidrug-resistant human hepatoma HepG2/ADM cells: *in vitro* and *in vivo* evaluation. Nanoscale Res. Lett. 6, 418 10.1186/1556-276X-6-418 21711951 PMC3211514

[B203] Zhang H. Yee D. Wang C. (2008a). Quantum dots for cancer diagnosis and therapy: biological and clinical perspectives. Nanomedicine (Lond.) 3, 83–91 10.2217/17435889.3.1.83 18393668

[B204] Zhang P. Ling G. Pan X. Sun J. Zhang T. Pu X. (2012). Novel nanostructured lipid-dextran sulfate hybrid carriers overcome tumor multidrug resistance of mitoxantrone hydrochloride. Nanomed. Nanotech. Biol. Med. 8, 185–193 10.1016/j.nano.2011.06.007 21704599

[B205] Zhang P. Ling G. Sun J. Zhang T. Yuan Y. Sun Y. (2011b). Multifunctional nanoassemblies for vincristine sulfate delivery to overcome multidrug resistance by escaping P-glycoprotein mediated efflux. Biomaterials 32, 5524–5533 10.1016/j.biomaterials.2011.04.022 21546082

[B206] Zhang T. Chen J. Zhang Y. Shen Q. Pan W. (2011c). Characterization and evaluation of nanostructured lipid carrier as a vehicle for oral delivery of etoposide. Eur. J. Pharm. Sci. 43, 174–179 10.1016/j.ejps.2011.04.005 21530654

[B207] Zhang X. Miao J. Dai Y. Du Y. Yuan H. Hu F. (2008b). Reversal activity of nanostructured lipid carriers loading cytotoxic drug in multi-drug resistant cancer cells. Int. J. Pharm. 361, 239–244 10.1016/j.ijpharm.2008.06.002 18586075

[B208] Zhang X. Xu X. Lam R. Giljohann D. Ho D. Mirkin C. (2011d). Strategy for increasing drug solubility and efficacy through covalent attachment to polyvalent DNA nanoparticle conjugates. ACS Nano 5, 6962–6970 10.1021/nn201446c 21812457 PMC3200554

[B209] Zhao P. Wang H. Gao H. Li C. Zhang Y. (2013). Reversal of multidrug resistance by magnetic chitosan−Fe_3_O_4_ nanoparticle−encapsulated MDR1 siRNA in glioblastoma cell line. Neurol. Res. 35, 821–828 10.1179/1743132813Y.0000000218 23651652

[B210] Zhou R. Mazurchuk R. Straubinger R. (2002). Antivasculature effects of doxorubicin containing liposomes in an intracranial rat brain tumor model. Cancer Res. 62, 2561–2566 11980650

[B211] Zhou Y. Shi L. Li Q. Jiang H. Lv G. Zhao J. (2010). Imaging and inhibition of multi-drug resistance in cancer cells via specific association with negatively charged CdTe quantum dots. Biomaterials 31, 4958–4963 10.1016/j.biomaterials.2010.02.053 20303165

[B212] Zingarelli B. Sheehan M. Wong H. (2003). Nuclear factor-[kappa] B as a therapeutic target in critical care medicine. Crit. Care Med. 31, S105–S111 10.1097/00003246-200301001-00015 12544984

